# A multicenter randomized controlled trial of medium‐chain triglyceride dietary supplementation on epilepsy in dogs

**DOI:** 10.1111/jvim.15756

**Published:** 2020-04-15

**Authors:** Benjamin A. Berk, Tsz H. Law, Rowena M. A. Packer, Annette Wessmann, Andrea Bathen‐Nöthen, Tarja S. Jokinen, Anna Knebel, Andrea Tipold, Ludovic Pelligand, Zoe Meads, Holger A. Volk

**Affiliations:** ^1^ Department of Clinical Science and Services Royal Veterinary College Hatfield UK; ^2^ BrainCheck.Pet Mannheim Germany; ^3^ Pride Veterinary Centre Derby UK; ^4^ Tierarztpraxis, Dr A. Bathen‐Nöthen Cologne Germany; ^5^ Faculty of Veterinary Medicine, Department of Equine and Small Animal Medicine Helsinki Finland; ^6^ Department of Small Animal Medicine and Surgery University of Veterinary Medicine Hanover Germany; ^7^ Department of Comparative Biomedical Sciences Royal Veterinary College Hatfield UK

**Keywords:** antiepileptic drug, antiseizure, canine, ketogenic diet, nutrition, refractory epilepsy

## Abstract

**Background:**

Medium‐chain triglyceride (MCT) enriched diet has a positive effect on seizure control and behavior in some dogs with idiopathic epilepsy (IE).

**Objective:**

To evaluate the short‐term efficacy of MCTs administered as an add‐on dietary supplement (DS) to a variable base diet to assess seizure control and antiseizure drug's (ASD) adverse effect profiles.

**Animals:**

Twenty‐eight dogs with International Veterinary Epilepsy Task Force Tier II (IVETF) level diagnosis of treated IE with 3 or more seizures in the last 3 months were used.

**Methods:**

A 6‐month multicenter, prospective, randomized, double‐blinded, placebo‐controlled crossover trial was completed, comparing an MCT‐DS with a control‐DS. A 9% metabolic energy‐based amount of MCT or control oil was supplemented to the dogs' diet for 3 months, followed by a control oil or MCT for another 3 months, respectively. Dogs enrolled in this study satisfied most requirements of IE diagnosis stated by the IVETF II level. If they received an oil DS or drugs that could influence the metabolism of the investigated DS or chronic ASD, the chronic ASD medication was adjusted, or other causes of epilepsy were found, the dogs were excluded from the study.

**Results:**

Seizure frequency (median 2.51/month [0‐6.67] versus 2.67/month [0‐10.45]; *P* = .02) and seizure‐day frequency were significantly (1.68/month [0‐5.60] versus 1.99/month [0‐7.42], *P* = .01) lower when dogs were fed MCT‐DS in comparison with the control‐DS. Two dogs were free of seizures, 3 had ≥50% and 12 had <50% reductions in seizure frequency, and 11 dogs showed no change or an increase in seizure frequency.

**Conclusions and Clinical Importance:**

These data show antiseizure properties of an MCT‐DS compared to a control oil and support former evidence for the efficacy of MCTs as a nutritive, management option for a subpopulation of drug‐resistant dogs with epilepsy.

**Abbreviations**
ASD: antiseizure drug
BHB: beta hydroxybutyric acid
C10: decanoic acid = capric acid
C8: octanoic acid = caprylic acid
DS: dietary supplement
IE: idiopathic epilepsy
ITTA: intention to treat analysis
IVETF II: International Veterinary Epilepsy Task Force Tier II
KBr: potassium bromide
LEV: levetiracetam
MBW: metabolic body weight
MCT: medium‐chain triacylglyceride
ME: metabolic energy
PB: phenobarbital
QoL: quality of life
RER: resting energy requirement
RVC: Royal Veterinary College
VAS: visual analog score

## 1 INTRODUCTION

Seizures and epilepsy are the most common neurological signs in dogs affecting an estimated 0.6%‐0.8%^1^, ^2^, ^3^ of the population. The various cellular pathophysiological mechanisms leading to epilepsy, characterized by unprovoked recurrent seizures, remain poorly understood. As a result, the enduring predisposition of having epileptic seizures can only be managed by medication for seizure suppression^4^ (antiseizure), instead of prevention of epilepsy^5^, ^6^ (antiepileptogenic). However, the chronic administration of antiseizure drugs (ASDs) presents difficulties in the balance between the beneficial seizure‐suppressive effects and undesirable drug‐related adverse effects.^7^ Around one‐third of dogs continue to seizure despite appropriately managed polypharmacotherapy.^8^, ^9^, ^10^ In addition, behavioral and cognitive comorbidities occur in dogs with epilepsy,^11^ such as anxiety^12^, ^13^ deficits in spatial memory,^14^ or cognitive function.^15^, ^16^ Seizures, drug‐related adverse effects along with changes to behavior and cognitive capabilities all contribute to the reduction in quality of life (QoL) for both dogs and their owners.^17^, ^18^, ^19^, ^20^, ^21^ Hence, new treatment strategies should address all aspects of epilepsy to improve the overall welfare of dogs and their owners.

Manipulation of nutrition has attracted increasing considerations as an alternative approach to impacting seizure activity and behavior.^22^, ^23^, ^24^ A cross‐sectional study revealed that over 60% of owners changed their dog's diet, after being diagnosed with idiopathic epilepsy (IE) in an attempt to improve seizure control and protect their dog from ASD‐related adverse effects.^25^ The influence of dietary modification on seizure control has been extensively studied in human medicine^26^, ^27^, ^28^, ^29^ and rodent epilepsy models,^30^, ^31^, ^32^, ^33^ showing antiseizure effects to varying degrees. Similarly, there are effects of nutritional management and diets in dogs with epilepsy^23^, ^24^, ^34^, ^35^, ^36^, ^37^, ^38^, ^39^ and paroxysmal dyskinesia.^40^, ^41^, ^42^, ^43^

**FIGURE 1:**
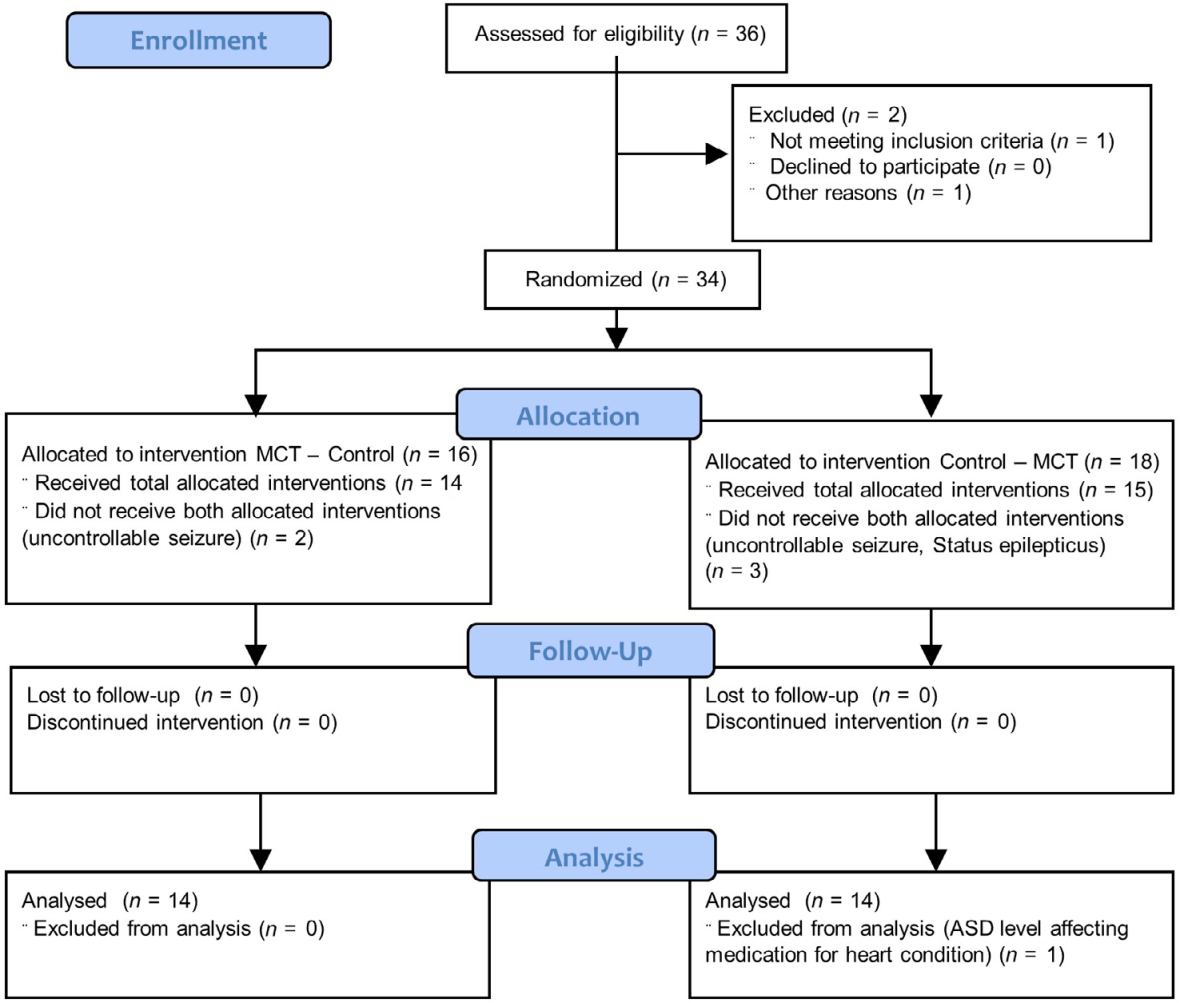
CONSORT flow diagram: flow diagram of the progress through the phases of this randomized clinical trial of 2 groups (ie, enrolment, intervention allocation, follow‐up, and data analysis)

A 6‐month prospective, randomized, double‐blinded, placebo‐controlled crossover dietary trial comparing a medium‐chain triacylglyceride (MCT) kibble diet (10% of the total formula calories from octanoic acid [C8], decanoic acid [C10], lauric acid [C12]) to a standardized placebo diet was completed in 21 chronically ASD‐treated dogs with IE (Figure 1).^24^ Consumption of a MCT diet resulted in statistically significant elevation of serum beta hydroxybutyric acid (BHB) concentrations and improvement in both seizure control^24^ and behavioral comorbidities.^23^ Although many owners add oil supplements or change their dogs' diet,^25^ it is currently unknown whether a MCT oil supplement might possess similar beneficial effects.^24^

The study hypothesis for the current study was that dietary supplementation with a commercially available MCT oil would reduce seizure frequency in dogs with IE and be well tolerated. Therefore, the aims of this clinical study were to investigate antiseizure effects and tolerability of MCT dietary supplement (MCT‐DS) in dogs with IE compared with a standardized control‐DS.

## 2 MATERIAL AND METHODS

This study was conducted in accordance with the guidelines of the International Cooperation of Harmonization of Technical Requirements for Registration of Veterinary Products GL9 Good Clinical Practices and the European Agency for the evaluation of Medical Products. This study was approved by the Royal Veterinary College (RVC) Clinical Research Ethical Review Board and ethical approval was granted (URN 2016 1558).

### 2.1 Study design

The study design has been published separately.^44^ In brief, the main study hypothesis was that MCTs administered as a DS would reduce seizure frequency. Thus, altered seizure frequency will serve as primary, measurable outcome variable to assess therapeutic effect. Changes in ASD adverse effects and quality of life of dogs with IE were assessed as a secondary outcome.

Based on the former research conducted 22 dogs completing the trial appeared sufficient to reach significance between groups.^24^, ^38^ A retrospective power analysis based on the former results of reducing seizure frequency when fed a MCT diet from 2.67 to 2.31/month in comparison with the placebo diet indicated that 22 dogs would be sufficient to reach a power of 0.95 (95%) at type I error rate of 0.05 (PASS V19.02, NCSS, Statistical Software, Kaysville, Utah). Considering in addition the usual dropout rate of around 30%, the aim was to recruit 36 dogs.

The study involved a 6‐month prospective, randomized, double‐blinded, placebo‐controlled, multicenter dietary crossover trial comparing the MCT‐DS to a standardized control oil DS for canine epilepsy. As a multicenter study, dogs were recruited over 6 study sites (Germany [Mannheim, Cologne, Hannover], Finland [Helsinki], United Kingdom [London, Derby]). Dogs enrolled onto this study were fed for 3 months (day 1 to day 90 ± 2) initially with either control‐DS or MCT‐DS alongside their normal diet, followed by a respective switch for another 3 months (day 90 to day 187 ± 2) to the other supplement alongside their normal diet. The first 7 days of dietary crossover, DS period 2 (day 90 to day 97 ± 2), were excluded from data collection and considered as the wash‐out period. Dogs were fed twice per day when each feed was supplemented with the respective DS‐oil representing 4.5% daily metabolic energy requirement for each dog (total 9% of metabolic energy [ME]). The DS allocations were block‐randomized and only available to the study nurse who was also the DS dispenser based at clinical investigation center at the RVC. Dog owners, investigators, other nurses, and statisticians involved were blinded throughout the study.

ASD treatment was not altered by any person involved at any point in this study. Per visit all dogs were evaluated on‐site. On each study visit relevant data were collected at the enrolment visit and start of the study (V1 = day 0), and at the end of each dietary intervention period (V2 = 90 ± 2 days, V3 = 187 ± 2 days) including general physical and neurological examination, seizure frequency (only generalized tonic‐clonic seizures were recorded); body weight; measurements of serum phenobarbital (PB), potassium bromide (KBr), or both concentrations as appropriate; dynamic bile acids; CBC; standard clinical serum chemistry; canine pancreatic lipase activity; adverse events; and visual analog scores (VAS) for ataxia, sedation, and QoL. Behavioral comorbidities and cognition have been measured but will be reported separately.

### 2.2 Dietary supplements

The experimental control and test products were commercially available DSs suitable for human consumption. The test oil was a MCT‐DS purified from palm and rapeseed oil containing 50%‐65% octanoic acid (C8) and 30%‐50% decanoic acid (C10) with 8.37 kcal/mL from 93% saturated fatty acids (End GmbH, Germany, Batch No. L 16M12). The control oil was colorless, extra‐virgin olive oil with 8 kcal per ml containing 11% saturated, 11% polyunsaturated, and 78% monounsaturated fat (Filippo Berio, Italy, Batch No LE194M04). Both oils were dispensed in brown bottles ensuring blinding of all involved. Prior to study setup, both oil supplements have been tested in a small cohort of healthy nonepileptic dogs (*n* = 19) on palatability, preference, side effects, and eating behavior confirming comparability.

The control and the MCT oil were stored at room temperature in a locked room at each test site. Owners were educated to keep the base diet consistent throughout the study. The quantity of DS‐oil given daily was calculated according to the metabolic weight, age, and neuter status of each dog individually and represented 9% isoenergetic requirement per day. The base diet was recorded, but not individually checked to fulfill nutritional requirements. A deviation of ±10% of the base diet consumption (kg) was allowed taking into consideration differences in activity level, physical condition, and to prevent gain or loss of body weight.

### 2.3 Case selection

Dogs were recruited by a 2‐step screening system through different media in the United Kingdom and Europe. An online prestudy questionnaire (SurveyMonkey) was completed for each dog, capturing all relevant data of epilepsy phenotype. Dogs were enrolled only when level of IE diagnosis^45^ satisfied most requirements stated by the International Veterinary Epilepsy Task Force Tier II IE diagnosis. Inclusion criteria required for enrolment onto this study were: age of seizure onset between 6 months and ≤12 years; weighing between 4 and ≤65 kg; unremarkable interictal neurological examinations for a dog on ASD; no clinically significant findings on CBC, unremarkable serum biochemistry, and dynamic bile acid results; unremarkable former cerebrospinal fluid analysis and magnetic resonance imaging scan; have had at least 3 generalized seizure episodes in the previous 3 months prior to enrolment; treated with at least 1 ASD and being classified as resistant to at least 1 of the ASD(s) given^6^ by responding with less than 50% reduction in seizure frequency. Dogs were excluded if they received an oil DS or drugs that could influence the metabolism of the investigated DS or chronic ASD. Dogs with other causes of epilepsy such as brain neoplasm, brain trauma, encephalitis, and meningitis; with chronic or acute renal, hepatic or cardiac disease; clinical history of pancreatitis or pancreatic insufficiency (exocrine pancreatic insufficiency, diabetes mellitus); with an acute or surgical condition at the time of enrolment; and bitches known or suspected to be pregnant or lactating were all excluded from the study. Dogs were enrolled as an individual experimental unit, and only 1 dog per household were enrolled onto this study. A unique study case number, consisting of a 3‐digit number (study side number‐study case number), ascending in a chronological order of enrolment, was assigned and used to identify each dog throughout the study. In total, 36 dogs were recruited onto this study.

## 3 SEIZURE DATA

Information from a standardized seizure diary per dietary intervention period was used to assess the short‐term effects of MCT‐DS on seizure control.^44^ At each study visit, the owners were repeatedly instructed, how to complete the seizure diary and each listed seizure event has been reevaluated by the owner's description. Only generalized seizures were counted toward the efficacy measurement and the primary outcome variable is the change of seizure frequency. Therapeutic success was defined as a significant difference (reduction) in 1 of seizure frequency (seizures/month), seizure days frequency per months, seizure cluster days frequency per months, seizure severity measured by number of episodes of cluster seizures or status epilepticus during a 3‐month period between the MCT, and control periods. Finally, each dog will be classified as a “MCT responder” if it experiences at least 50% reduction in seizure frequency between both dietary periods.

### 3.1 Visual analog score

Visual analog score for ataxia, sedation and overall QoL was completed by the owner at each study visit. The owner was asked to mark a measurement line ranging from 0 to 100 mm (0%‐100%) perpendicularly with a secondary intersecting line that presented best the subjective severity (0% [better, than normal] to 100% [cannot be worse]). A perpendicular line at 0 mm, baseline (B), represented “asymptomatic/normal” and at 100 mm represented either “ataxia so severe dog is unable to walk” or “sedation to the extent dog only sleeps” or “QoL is so poor euthanasia is requested,” respectively. The length from 0 to the perpendicular line in mm was compared between periods and expressed as a percentage value (0%‐100%, negative = improvement, positive = deterioration).

### 3.2 Owner‐based epilepsy disease‐specific quality of life aspects (EpiQoL)

In addition to the QoL VAS, the impact from dietary supplementation on epilepsy and its treatment on the QoL of the dogs and their owners was assessed at the end of each dietary intervention period. A questionnaire based on the carer's perception of their dogs and their own QoL (epilepsy disease‐specific quality of life list of key questions = EpiQoL) was used to study QoL aspects and adverse effect profiles associated with MCT‐DS use.^17^, ^18^

### 3.3 Ketone body measurements

Preprandial and postprandial BHB concentrations were analyzed on each visit day (V1‐V3). Preprandial blood samples were collected at initial presentation in the morning after fasting for at least 12 hours. Postprandial samples were taken 2 hours after consumption of their usual diet including the prescribed amount of DS per period. Both samples were stored using clotting activator dipotassium ethylenediaminetetraacetic acid‐containing (with serum‐separation gel) polypropylene blood‐collection tubes and plain polypropylene blood‐collection tubes (International Scientific Supplies Ltd, Bradford, UK). Blood samples were allowed to clot and shipped for subsequent analysis for BHB concentrations at 1 of the local laboratories (Diagnostic Laboratory Services, RVC, UK; IDEXX Laboratories, Inc., Ludwigsburg, Germany).

### 3.4 Adverse events and concomitant treatments

Any adverse, abnormal, or both events occurring after enrolment were reported to the investigator and documented (Table S1). Seizure occurrences throughout the study were not classified as adverse events. Concomitant treatments administered throughout the study were recorded detailing indication, products used and strength, dose and length of treatments administered (Table S2). Concomitant changes of ASD medication or dosages throughout the study led to study exclusion.

### 3.5 Statistical analysis

Seizure frequency, defined as the number of seizures per month, and seizure day frequency, defined as the number of days in which seizures occurred within a month, were compared between DS periods. Monovariate comparisons between the MCT‐DS and control DS groups were conducted using match‐paired Student's *t* tests for parametric data and Wilcoxon matched‐pairs signed rank test for nonparametric data (GraphPad Prism; STATCON GmbH, Witzenhausen, Germany). The presence of cluster seizures was compared between DS periods using the McNemar test. The relationship between 2 continuous variables, such as seizure frequency and body weight, were analyzed using Pearson's correlation coefficient analysis. All comparisons were 2‐sided, and *P* < .05 was considered significant. Nonparametric data are presented as median (25th to 75th percentile), and parametric data are represented as mean values and SD values. Considering potential‐fixed (type of DS) and random effects (treatment order, study center location) of the study design on seizure occurrence a multivariate analysis was performed using a linear mixed model.

## 4 RESULTS

### 4.1 Study population

The study protocol was completed by 28 of the 36 recruited dogs, which included 18 different breeds at 5 different study sides in 3 different countries (UK [RVC, N = 12, 43% and Pride Veterinary Centre, N = 4, 14%], Germany [Tierarztpraxis Dr. A. Bathen‐Nöthen, N = 4, 14%; University of Veterinary Medicine Hannover, N = 3, 11%; and Tierarztpraxis Strassenheim, N = 4, 14%], and Finland [University of Helsinki, N = 1, 4%]). One dog of each of the following breeds were included unless otherwise specified: Australian shepherd, Basset Griffon Vendeen (Petit), Bernese Mountain dog, Border Collie (N = 3), Border Terrier, Cairn Terrier, Chihuahua, Cross breeds (N = 8), Dogue de Bordeaux, German shepherd, golden retriever, Hungarian Vizsla, Keeshond, Labrador retriever, Samoyed (N = 2), Slovakian Rough Haired Pointer, Staffordshire Bull Terrier, Whippet (Table S3). The study population consisted of 16 males, of which were 12 neutered and 4 were intact, and 12 females, of which 10 were neutered and 2 intact. The population of dogs was on average 5.46 ± 2.61 years of age and weighed 25.6 ± 13.4 kg at start of trial.

### 4.2 Antiseizure medication regimes

All 28 dogs were drug resistant to at least 1 of the ASD given and defined as partial ASD‐nonresponders with less than 50% reduction in seizure frequency. Two dogs received imepitoin only, the rest were under chronic ASD combination treatment (N = 26; Table S4). Thirty‐two percent (N = 9) of the 28 dogs received a second and 61% (N = 17) a third ASD. Twenty‐five (89%) of the 28 dogs received PB as ASD. The predominant combination treatment in 40% of the cases was PB, KBr, and levetiracetam (LEV; N = 14). For the acute treatment of cluster seizures, 12 owners used rectal diazepam of which 3 owners also used LEV as pulse treatment. No alterations occurred for the acute or chronic drug treatment regimens between the control or MCT‐DS period.

### 4.3 Main dietary regime and dietary supplementation

The majority of dogs (82%, N = 19) were fed dry food as a single diet (46%) or in combination with wet food (11%), raw feed (14%), or home‐cooked feed (11%). The remaining 18% were fed self‐cooked food or raw feed. Independent of the predominant feeding type, chicken was in 50% of all dogs (N = 14), the main source of meat, followed by beef (29%), salmon (20%), and lamb (14%). Cereals (N = 12 [43%], wheat, maize, rice, soya), potatoes (N = 6, 22%), other vegetables (N = 8 [29%], peas, carrots), or fruits (N = 2, 8%) were other main components in their feed. In 68% (N = 19) of participating dogs, it was possible to calculate an average dry matter (DM)‐based composition of the baseline diet from company provided analytical data mirroring a low fat and low protein diet (crude protein 21.8%, crude fat 11.75%, crude fibers 3.9%, crude ash 5.96%, calcium 1.18%, phosphorus 0.78%) (Table S5). While 5 dogs (18%) were on a veterinary prescription diet with low‐fat content (<12% crude fat in 100 g DM), only 2 dogs were fed complete carbohydrate‐free regime with raw meat only. Fifteen out of 28 dogs received as first DS the control‐DS, while 14 got the MCT‐DS first, subsequently followed by the other DS at crossover. The average administered oil amount was 7.03 ± 2.68 mL of MCT‐DS or 7.01 ± 2.69 mL of control‐DS split into 2 portions a day.

### 4.4 Effects on seizure frequency, seizure day frequency, and severity of seizures

**FIGURE 2:**
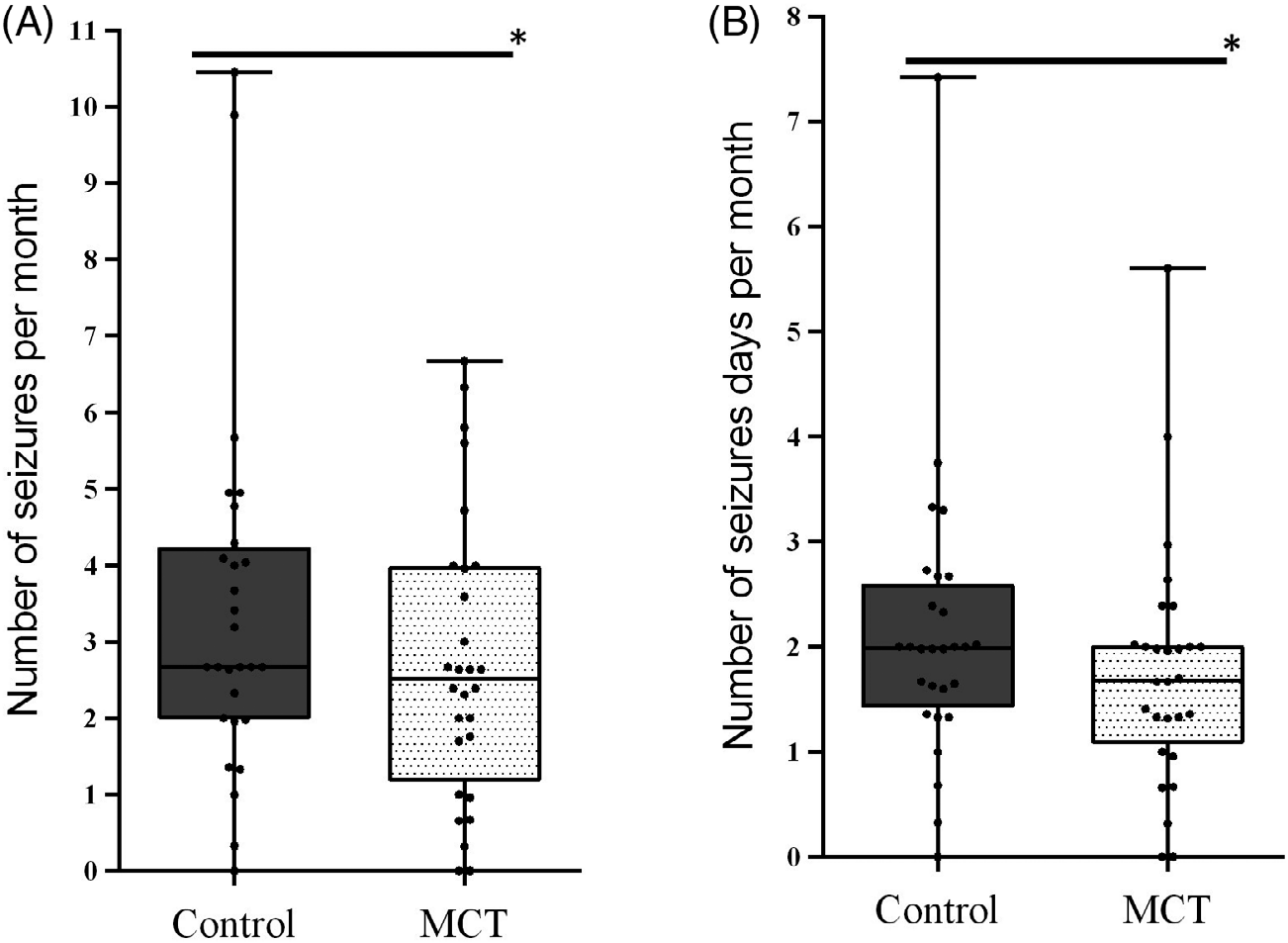
Effect of the medium‐chain triglyceride dietary supplement (MCT‐DS, light pointed) on: A, seizure frequencies per month and, B, seizure days per month compared with the control dietary supplement (control‐DS, dark‐gray; N = 28). Significant reductions in the A, number of seizures per month (*P* = .02) and B, seizure days per month (*P* = .02) during the MCT‐DS phase in comparison with the control‐DS was found. Data are shown as box‐and‐whisker plots (central lines of the box represent the median, lower, and upper limits of the box represent the 25th and 75th percentiles and whiskers represent the minimum and maximum). Two‐sided Wilcoxon's matched‐pairs' rank tests were used to compare control and MCT‐DS groups. *P < .05

Seizure frequency was significantly lower when dogs were fed MCT‐DS (median 2.51/month, 0‐6.67/month) in comparison with the control‐DS (2.67/month, 0‐10.45/month; *P* = .02, Figure 2). During the MCT‐DS period, 2 dogs achieved were seizure free (100% reduction), 3 dogs had a 50% or greater reduction in seizure frequency (58%, 50%‐66%) and 12 dogs had an overall reduction in seizure frequency (21%; 6%‐42%). Eleven dogs showed no response to MCT‐DS with no change (N = 3) or an overall increase in seizure frequency per month (N = 8, 8%; 2%‐33%) during this period (Table S6).

Seizure day frequency was also significantly lower when dogs were on the MCT‐DS (1.68/month, 0‐5.60/month versus 1.99/month, 0‐7.42/month, *P* = .01, Figure 2) with over 75% of the trial population achieving reduction in seizure days during this period. During the MCT‐DS period 2 dogs were free of seizures (100% reduction) and 15 dogs had an overall reduction (24%, 11%‐40%) in seizure day frequency. In comparison, under control‐DS none of the dogs were seizure free or had an overall reduction of more than 50%, but 17 dogs (61%) had an increase of seizure frequency. Eleven dogs showed no response to MCT‐DS with either no change (N = 5) or an overall increase in days per month with seizure occurrence (N = 6, 33%, 15%‐42%). More than 1 seizure in a day was reported in 75% of the study population during the control‐DS period and only 57% of the study population during the MCT‐DS period (McNemar, *P* = 0.06), but this was not significantly different. No other associations were identified (Table S7).

Potential biases such as treatment order (MCT—control, control—MCT) or the effect of the study center were considered in monovariate comparison and multivariate statistical mixed model.

On monovariate comparison, the average reduction rate in seizure frequency appeared not significantly different for treatment order or study center; and no association of the supplementation group with treatment order (*r* = .1793, *X* ^2^ = 0.03216, *P* = .36) or study center (*r* = 0.1629, *X* ^2^ = 0.02653, *P* = .41) has been identified.

Using the type of DS as fixed and treatment order and study location as random effects in a multivariate analysis as linear mixed model, seizure frequency (*P* = .02) and seizure day frequency (*P* = .01) appeared significantly reduced during MCT treatment period compared to control.

### 4.5 Effects on body weight, serum antiepileptic drug concentration, CBC, clinical chemistry, and blood ketone concentrations

There was a significant reduction in serum concentrations of PB during MCT‐DS consumption when compared to the control‐DS period (30.04 ± 7.81 [13.1‐44.3] g/mL versus 32.78 ± 7.91 [17.3‐49.2] g/mL, *P* = .01). Alkaline phosphatase activity was also lowered during the MCT‐DS study period (163.5 U/L [83.25‐1578] versus 185.5 U/L [97.5‐2541], *P* = .03). However, KBr serum concentrations (1.706 ± 0.45 mg/mL [0.66‐2.54] versus 1.728 ± 0.59 mg/mL [0.59‐2.66], *P* = .79), pancreas lipase activity (90 μg/mL [30‐146.3] versus 83.5 μg/mL [30‐150.8]), and weight (26.79 ± 13.32 versus 26.47 ± 13.16 kg, *P* = .98) were not significantly different between control‐DS and MCT‐DS periods, respectively.

There were significantly higher concentrations of BHB in both pre and postprandial blood samples during the MCT‐DS period when compared to the control‐DS period (preprandial: 0.07 ± 0.055 versus 0.06 ± 0.048 mmoL/L [*P* = .03]; postprandial: 0.059 ± 0.037 versus 0.049 ± 0.028 mmoL/L [*P* = .01]), respectively. However, the differences between preprandial and postprandial BHB blood concentrations within DS periods were not significantly different ([ΔMCT‐DS: 0.01 versus Δcontrol‐DS: 0.01] mmol/L; *P* = .67). All other CBC, biochemistry, glucose, and bile acids stimulation results were not significantly different between the DS groups.

### 4.6 Effects on VAS and adverse effect profiles of antiepileptic drug medication

**FIGURE 3:**
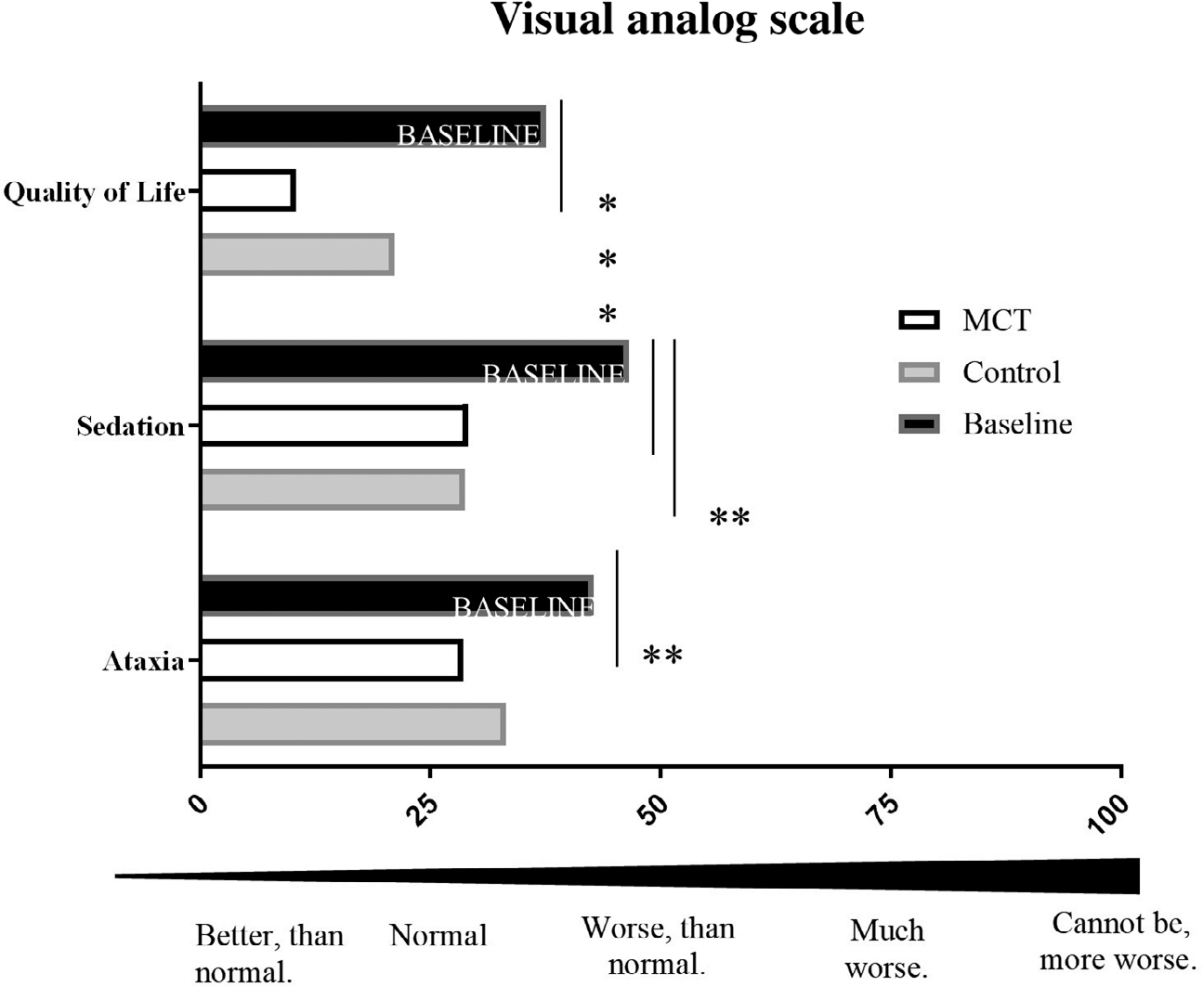
Effects of the medium‐chain triglyceride dietary supplement (MCT‐DS) on ataxic gait, sedation and quality of life (QoL). Visual analog score was compared per dietary supplementation period to baseline records at enrolment visit to assess antiseizure drug‐related adverse effect profiles in the course of the trial. The owner was asked to draw a secondary intersecting line perpendicular to the line of measurement that best represented the subjective severity (0 [better, than normal] to 100% [cannot be worse]). The figure shows the owner reported perception and in percentage normalized grading of each aspect illustrated in a column bar graph. The MCT‐DS intake resulted in significant improvement of ataxia (*P* = .003), sedation (*P* = .002), and QoL (*P* = <.001) of the population (N = 28)

There were significant differences in the VAS compared to baseline. During the MCT‐DS period it was reported that dogs were less sedated (B: 47% ± 28% versus MCT: 29%±18%, *P* = .002), less ataxic (B: 43% ± 28% versus MCT: 29% ± 25%, *P* = .003) than baseline and had an overall better owner reported QoL (B: 38% ± 21% versus MCT: 10% ± 14%, *P* ≤ .001). Apart of sedation (B: 47% ± 28% versus control [C]: 29% ± 21, *P* = .007), there were no other significant differences in VAS comparisons between MCT and control‐DS or both with baseline (Figure 3).

Similarly, in the EpiQoL questionnaire, the owner's perceptions between both dietary periods (MCT‐DS, control‐DS) and the baseline (B) have been compared. Owners reported that during the MCT‐DS study period they were less “bothered” by their dog's mental status (1‐5 [not at all bothersome‐extremely bothersome], B: 2 [1‐5] versus MCT: 2 [1‐2], *P* = .03) and that their dog had a less severely ataxic gait (1‐5 [very mild‐very severe]; B: 3 [1‐4]versus MCT: 2 [1‐3], *P* = .003; C: 3 [2‐4 ]versus MCT: 2 [1‐3], *P* = .002). In addition, the owners reported that they better tolerated the ASD adverse effects seen in their dogs (1‐5 [strongly agree‐strongly disagree]; B: 3 [2‐4] versus MCT: 2 [1‐3], *P* = .004; C: 3 [3‐4] versus MCT: 2 [1‐3], *P* = .003) than before or in comparison to the control oil.

### 4.7 Adverse events and disease‐related death

Observation about any unfavorable, unintended health abnormality occurred after enrolment, regardless it was considered as MCT‐DS‐ or control DS‐related event, was reported to the investigator and documented (Table S1). Independent of the administered DS, no adverse event occurred more than once during 1 dietary period or was predominantly found associated to the type of DS. Seizure occurrences and disease‐related death throughout the study were not classified as adverse events and considered as sudden unexpected death in epilepsy.^6^, ^35^, ^46^

### 4.8 Withdrawn and excluded dogs

Eight further dogs were recruited but did not complete the study, of which 2 were excluded at visit 1 prior to DS allocation. One dog had a significant increase in canine pancreatic lipase activity, while the other dog received chronic administration of cannabidiol. Furthermore, 1 dog was excluded after completion of the trial and 5 were withdrawn during the trial. For the dogs which withdrew, 2 dogs were on the MCT‐DS period and 4 were on the control‐DS period at time of withdrawal. Two dogs, withdrawn during the MCT‐DS period, were euthanized before study completion due to uncontrollable cluster seizures and owner‐directed postictal aggression. On the other hand, 2 dogs were euthanized due to uncontrollable cluster seizures or status epilepticus during the control‐DS period. One dog was withdrawn, during the control‐DS period, as the owner requested to alter their ASD treatment due to uncontrollable cluster seizures. One dog was excluded from subsequent data analysis after completion of the trial as this dog had been diagnosed with pulmonary hypertension and dilatative cardiomyopathy, from which alterations in ASD clearance and elimination were expected due to chronic furosemide consumption.

## 5 DISCUSSION

**FIGURE 4:**
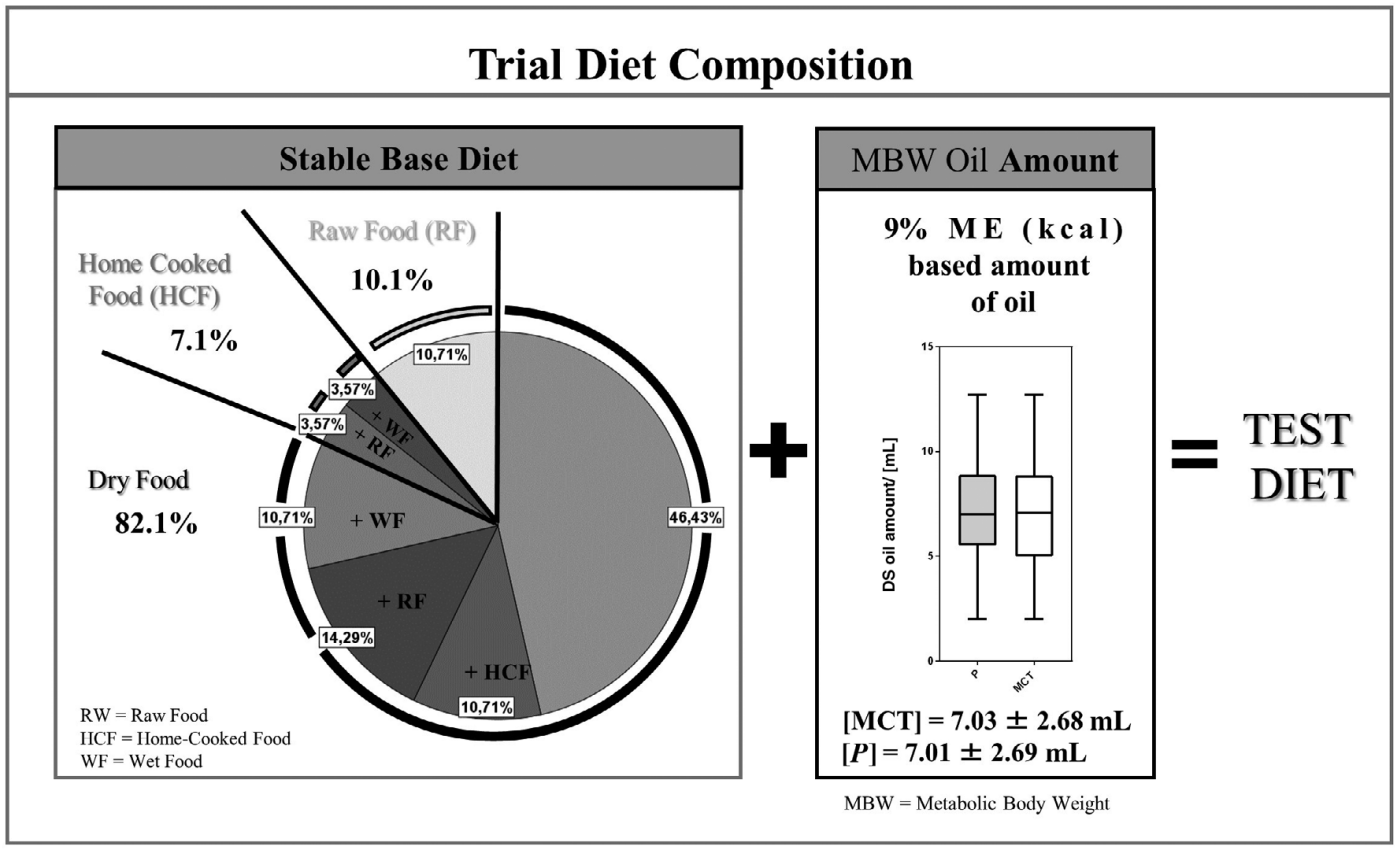
Trial diet composition: the figure summarizes the distribution of the different main feeding regimes within the study population (N = 28). The in average calculated oil amount based on 9% of the ME requirement was 7.03 (SD 2.68) mL of MCT‐DS or 7.01 (SD 2.69) mL of control‐DS split into 2 portions a day added to the base diet. The oil amount was calculated from the patients metabolic energy requirement derived from the metabolic body weight (RER = 70 [10 kg]^3/4^, ME = RER × factor, life stages corresponding factors used to estimate daily energy needs for dogs). DF, dry food; HCF, home‐cooked food; MBW, metabolic body weight; MCT‐DS, medium‐chain triglyceride dietary supplement; ME, metabolic energy; RER, resting energy requirement; RW, raw food; WF, wet food

The objective of this clinical trial was to evaluate the antiseizure efficacy and tolerability of an MCT oil in dogs with IE chronically treated with standard ASDs. Twenty‐eight dogs with epilepsy were included in the 6‐month randomized, placebo‐controlled, crossover prospective trial. The supplementation of commercially available MCT oil, containing C10 and C8, to a stable, individually variable base diet was well tolerated and decreased both seizure frequency and seizure day frequency compared to control oil (Figure 4).

Epidemiological studies suggest differences between the intrinsic severity of epilepsy among individuals linked to their response to medication and the long‐term therapeutic success. In addition, individual seizure‐associated alteration on pharmacodynamics and kinetics of ASD has been discussed.^47^ This might explain that in human patients only around 10% of the overall cohort are seizure free when a 2nd ASD is added to the treatment and less than 4% for any additional ASD given.^48^ Some veterinary studies report an even lower seizure freedom when a 2nd or 3rd ASD is given.^49^ When more than 50% reduction in seizure frequency is used as definition of ASD response, usually a third of the reported population in the literature will respond to the 2nd ASD.^8^, ^10^, ^50^ In the current study, all 28 dogs in this trial were classified as resistant to at least 1 ASD and suffered from at least 1 seizure per month at trial start. Two dogs received 1 ASD (impetoin), while most dogs were on 2 (N = 9) or 3 drugs (N = 17) as antiseizure combination therapy. The overall reduction in seizure frequency between treatment groups was small and that only a small cohort of animals was seizure free or had more than 50% reduction in seizure frequency. Two dogs achieved seizure freedom, 3 additional dogs had ≥50% and 12 had <50% reductions in seizure frequency and 11 dogs showed no change or an increase in seizure frequency. The response to MCT treatment was better in the study reported by Law et al^24^ with 14% becoming seizure free and 48% of the dogs having more than 50% reduction in seizure frequency. In both studies MCTs were very well tolerated and in the current study even an improvement is the ASD adverse‐effect profile was noted. Owner‐reported improved level of sedation and ataxia and perceived QoL during the MCT‐DS period compared to baseline. This could be at least in parts explained by a small but significant reduction in PB serum concentrations when dogs were on the MCT‐DS. There were no significant differences in body weight, bile acids, cholesterol, pancreatic lipase activity, glucose, and KBr serum concentrations between MCT‐DS and control DS periods. MCT‐DS as dietary management appears safe and might be considered as an option for some individual dogs.

Consumption of the MCT‐DS reduced PB serum concentrations by approximately 8% and reduced liver enzyme alkaline phosphatase activity by 10% when compared to the control‐DS. Similar results were not observed when dogs with IE were fed a complete kibble diet enriched with MCTs.^24^, ^38^ The pharmacokinetic properties of ASDs have been shown to be influenced by other medications,^51^ diets,^52^, ^53^, ^54^, ^55^, ^56^, ^57^ or both, whereby interactions might manifest via amplification of liver metabolism. Maguire et al showed in 2000 that dietary protein, fat, or both restriction led to significantly decreased volume of distribution and half‐life, and increased clearance and elimination rates of PB.^57^ It is of interest to note that a fat‐ and protein‐restricted diet resulted in increased serum alkaline phophatase activity.^57^ The results presented in this study show that MCT oil and its respective metabolic constituents may also affect the absorption and excretion rates of PB.^58^, ^59^ Furthermore, it has been demonstrated that PB, when consumed with food, is not absorbed as well as in a fasting state and decreases the bioavailability.^54^ MCT oil‐induced perturbations on PB absorption could lead to decreased liver enzyme activity, such as alkaline phosphatase activity, and partly improved adverse effect profiles.^7^, ^60^

PB induces the expression of cytochrome P450 genes and increase liver enzyme activities,^61^, ^62^, ^63^ and CYP152 family P450s to oxidatively decarboxylate fatty acids and induce expression levels.^64^ MCT are metabolized by some CYP4 isoforms, which is a family of P450 enzymes.^65^ The reduced PB concentration associated with reduced alkaline phosphatase enzyme activities might therefrom also result from MCT provoked higher cytochrome p450 activities in the liver.^66^ Further in vitro studies are needed to confirm this observation and link it to potential metabolic pathways.

Although PB serum concentrations were significantly lower during MCT consumption, the relative reduction might or might not be clinically relevant for the improvements in both seizure control or owner‐perceived adverse effects shown in this study.^67^, ^68^ The supplementation of MCT oil described in this trial resulted in a significant reduction of ASD adverse effects, which has not been previously reported. Owners reported an improvement in ataxia and sedation which mighty have contributed to owners also thinking that their dog's QoL has improved during the MCT‐DS period compared to baseline. These changes suggest a better tolerability of ASD. Antiepileptic and aforementioned pharmacokinetic properties of MCT oil supplementation may act in a synergistic manner, leading to less adverse effects for epileptic dogs with improved seizure control.

Preprandial and postprandial blood BHB concentrations were both significantly elevated in this trial, when dogs consumed the MCT‐DS in comparison to the control‐DS, which is in accordance with dogs fed an MCT‐enriched kibble diet.^24^ In a Drosophila invitro model, BHB reduces seizure‐like neuronal activity, which is thought to be mediated via K_ATP_ channel activity and GABA_B_ signaling inhibition.^69^ In a kainic acid rat seizure model intraperitoneal BHB administration prolonged the onset of induced seizures. In addition, neuronal cell death was alleviated and linked to protective effects.^70^ More recent in vitro studies also suggest that BHB exerts beneficial neuroprotective functions by way of gene regulation and promotion of brain‐derived neurotrophic factor expression.^71^ Other than seizure suppressing effects, elevated blood BHB concentrations have anxiolytic effects in lab rats.^72^ In humans receiving a ketogenic diet (KD), the increase in blood BHB concentrations and other ketone bodies is considered to play a crucial role for improved seizure control.^28^, ^30^, ^73^ Here, the majority of dogs received over 50% of their daily calorie intake from carbohydrates (nitrogen‐free extract: 56.59%; from cereals, potatoes, or other vegetables), fat (11.75%), and protein (21.8%). This macronutrient profile is in contradiction to the common KDs in human medicine which are usually of a high‐fat, low‐carbohydrate composition.^74^ Therefrom, the results of this study demonstrate also that MCT supplementation with a non‐KD base diet can enhance BHB production. However, the dogs did not become ketotic, as observed in humans under KD diet. Accordingly, it might be suggestive that the rapid onset of antiseizure properties in dogs consuming the MCT‐DS might be governed more by the direct effect of certain MCTs, rather than the downstream metabolites of BHB.

The MCT‐enriched kibble diet reported by Law et al in 2015 contained medium‐chain fatty acids C8, C10, and C12 which was the only compositional difference to the control diet.^24^ C10 not only acts as a noncompetitive AMPA receptor antagonist resulting in direct inhibition of excitatory neurotransmission, and thus exerts an anticonvulsant effect,^75^ but also has synergistic effects with a newly introduced ASD in human medicine.^76^ Furthermore, C10 on its own might be able to modulate mitochondrial proliferation via PPARγ receptor^77^ and improve brain mitochondrial energy homeostasis in a dose‐dependent manner.^78^ An enhanced anticonvulsant effect is observed in mice under seizure provocation by intravenous administrated pentylenetetrazol, when C10 and C8 was orally coadministered.^79^ In multiple vitro seizure and status epilepticus models, the inactive unbranched compound C8 does not to play a direct role in provoking antiepileptic properties.^80^, ^81^ However, methyl‐branched derivatives of C8 enhance seizure control and neuroprotection.^82^ Thus far, the available scientific evidence indicates that C10 may be more relevant than C8 with regard to active interaction with cellular targets that can explain antiseizure effects from MCT consumption.^31^, ^83^, ^84^

The dogs' baseline diets in this study have not been controlled to ensure a balanced nutrient supply as they varied between the trial participants and therefore must be considered a limitation of this diet trial study. In addition, the original MCT content cannot be evaluated in the baseline diet. However, the dietary access to MCTs is limited in the usual diet of dogs. Average sources used in production of commercial foods or the composition of a home‐made or raw‐feed normally contain relatively low concentrations or no MCTs.^85^, ^86^ In commercial pet food, MCTs are typically not added to diets due to reports on decreased food palatability.^87^ Therefrom, a negligible impact on the results of the baseline on this trial could be expected. Likewise, because MCTs are so rarely used in commercial pet food, its supplementation or integration may easily offer more options to manage chronic neurological diseases and should be explored further in the future.

The oral tolerability of MCTs in dogs has been widely explored with controversial findings and the occurrence of gastrointestinal signs or palatability issues were more likely.^87^, ^88^, ^89^, ^90^, ^91^ The percentage of metabolic energy chosen as amount of MCT‐DS was orientated on the previous study from Law et al in 2015, but also tolerance and palatability findings from other studies,^87^, ^88^, ^89^, ^90^, ^91^ but also recommendation to meet nutritional guidelines established by the Association of American Feed Control Officials and the National Research Council.^92^ In this trial, adverse events have not been found in higher frequency or predominantly associated to 1 type of DS in this trial. Thus, the seen adverse events were considered as not provoked by the dietary interventions of this study and as naturally occurring diseases.

One relevant limitation of this clinical study could be the study center effect. However, as the number in each of the centers was rather small and each dog experienced at each visit the same scenario, a center effect was expected to be small and was not identified on statistical analysis. We were also not able to identify a treatment order bias. In addition, the findings of the current trial confirm earlier findings in a single center study.^24^ Another limitation of the analysis was that an intention to treat analysis (ITTA) was not conducted. Normally, ITTA is an analysis approach in which all recruited patients are evaluated as block randomized, regardless of the dietary intervention they actually received.^93^ However, as in randomized clinical trial with epileptic patients, the dropout rate appears to be higher, especially in the beginning, all of the 8 dogs dropped out in the first leg of the study. Therefore, an ITTA would not be appropriate.

In summary, this trial provides additional evidence for the use of MCT oil as a DS to a stable base diet as a potential therapeutic management option for dogs with epilepsy. Although the overall significant statistical effect was small, some dogs had a good response. Future studies are needed to test, if MCT can be clinically more significant in drug‐naive dogs or dogs with less drug‐resistant epilepsy. The result presented by this study further emphasizes the potential implications of dietary management for both human and canine drug‐resistant epilepsy. The effectiveness and efficacy of the MCTs on epilepsy should be further investigated involving a pragmatic approach with larger longer‐lasting randomized controlled trials and inclusion of an intention‐to‐treat analysis.

## CONFLICT OF INTEREST DECLARATION

Authors declare no conflict of interest.

## OFF‐LABEL ANTIMICROBIAL DECLARATION

Authors declare no off‐label use of antimicrobials.

## INSTITUTIONAL ANIMAL CARE AND USE COMMITTEE (IACUC) OR OTHER APPROVAL DECLARATION

The study protocol and design were approved by the Clinical Research Ethical Review Board (CRERB) and ethical approval has been granted (URN 2016 1558). The data collected in this trial are collated and stored at the Royal Veterinary College in London (RVC). Data were anonymized as appropriate, and only used for analysis. All dog's personal information was held and used in accordance with the GDPR 2018 and will not be disclosed to any unauthorized person or body.

## HUMAN ETHICS APPROVAL DECLARATION

Authors declare human ethics approval was not needed for this study.

## Supporting information


**Table S1**. Adverse events recorded for dogs during the dietary supplementation with the control and MCT‐DS
**Table S2**. Concomitant treatment
**Table S3**. Age, weight, sex, neuter status, breed and base diet composition of the all dogs
**Table S4**. Antiepileptic drug medication regimes of each individual dog included in this study
**Table S5**. Trial diet composition—the average macronutrient profile of the base diet
**Table S6**. Overview of seizure frequency per month and seizure day frequency per month in each dog during the control DS and MCT‐DS
**Table S7**. Correlation coefficient analysis between seizure frequency reduction and seizure day frequency reduction with age, weight and BHB concentrations

## References

[1] Erlen A , Potschka H , Volk HA , Sauter‐Louis C , O'Neill DG . Seizure occurrence in dogs under primary veterinary care in the UK: prevalence and risk factors. J Vet Intern Med. 2018;32:1665‐1676. 30216557 10.1111/jvim.15290 PMC6189390

[2] Kearsley‐Fleet L , O'Neill DG , Volk HA , et al. Prevalence and risk factors for canine epilepsy of unknown origin in the UK. Vet Rec. 2013;172:338. 23300065 10.1136/vr.101133

[3] Heske L , Nodtvedt A , Jaderlund KH , et al. A cohort study of epilepsy among 665,000 insured dogs: incidence, mortality and survival after diagnosis. Vet J. 2014;202:471‐476. 25457266 10.1016/j.tvjl.2014.09.023

[4] Bhatti SF , De Risio L , Munana K , et al. International veterinary epilepsy task force consensus proposal: medical treatment of canine epilepsy in Europe. BMC Vet Res. 2015;11:176. 26316233 10.1186/s12917-015-0464-z PMC4552371

[5] Scheffer IE , Berkovic S , Capovilla G , et al. ILAE classification of the epilepsies: position paper of the ILAE Commission for Classification and Terminology. Epilepsia. 2017;58:512‐521. 28276062 10.1111/epi.13709 PMC5386840

[6] Berendt M , Farquhar RG , Mandigers PJ , et al. International veterinary epilepsy task force consensus report on epilepsy definition, classification and terminology in companion animals. BMC Vet Res. 2015;11:182. 26316133 10.1186/s12917-015-0461-2 PMC4552272

[7] Charalambous M , Shivapour SK , Brodbelt DC , Volk HA . Antiepileptic drugs' tolerability and safety—a systematic review and meta‐analysis of adverse effects in dogs. BMC Vet Res. 2016;12:79. 27206489 10.1186/s12917-016-0703-y PMC4875685

[8] Munana KR . Management of refractory epilepsy. Top Companion Anim Med. 2013;28:67‐71. 24070684 10.1053/j.tcam.2013.06.007

[9] Podell M , Fenner WR . Bromide therapy in refractory canine idiopathic epilepsy. J Vet Intern Med. 1993;7:318‐327. 8263851 10.1111/j.1939-1676.1993.tb01025.x

[10] Podell M . Antiepileptic drug therapy. Clin Tech Small Anim Pract. 1998;13:185‐192. 9775509 10.1016/S1096-2867(98)80040-6

[11] Jokinen TS , Tiira K , Metsähonkala L , et al. Behavioral abnormalities in Lagotto Romagnolo dogs with a history of benign familial juvenile epilepsy: a long‐term follow‐up study. J Vet Intern Med. 2015;29:1081‐1087. 25945683 10.1111/jvim.12611 PMC4895370

[12] Packer RM , De Risio L , Volk HA . Investigating the potential of the anti‐epileptic drug imepitoin as a treatment for co‐morbid anxiety in dogs with idiopathic epilepsy. BMC Vet Res. 2017;13:90. 28388948 10.1186/s12917-017-1000-0 PMC5383962

[13] Shihab N , Bowen J , Volk HA . Behavioral changes in dogs associated with the development of idiopathic epilepsy. Epilepsy Behav. 2011;21:160‐167. 21531631 10.1016/j.yebeh.2011.03.018

[14] Winter J , Packer RMA , Volk HA . Preliminary assessment of cognitive impairments in canine idiopathic epilepsy. Vet Rec. 2018;182:633. 10.1136/vr.104603 29700175

[15] Packer RMA , McGreevy PD , Pergande A , et al. Negative effects of epilepsy and antiepileptic drugs on the trainability of dogs with naturally occurring idiopathic epilepsy. Appl Anim Behav Sci. 2018;200:106‐113.

[16] Packer RMA , McGreevy PD , Salvin HE , et al. Cognitive dysfunction in naturally occurring canine idiopathic epilepsy. PLoS One. 2018;13:e0192182. 29420639 10.1371/journal.pone.0192182 PMC5805257

[17] Wessmann A , Volk HA , Packer RM , et al. Quality‐of‐life aspects in idiopathic epilepsy in dogs. Vet Rec. 2016;179:229. 27329504 10.1136/vr.103355

[18] Wessmann A , Volk HA , Parkin T , Ortega M , Anderson TJ . Evaluation of quality of life in dogs with idiopathic epilepsy. J Vet Intern Med. 2014;28:510‐514. 24612035 10.1111/jvim.12328 PMC4858018

[19] De Risio L , Freeman J , Shea A . Evaluation of quality of life of carers of Italian spinoni with idiopathic epilepsy. Veterinary Record Open. 2016;3:e000174. 27403328 10.1136/vetreco-2016-000174 PMC4932266

[20] Lord LK , Podell M . Owner perception of the care of long‐term phenobarbital‐treated epileptic dogs. J Small Anim Pract. 1999;40:11‐15. 10092036 10.1111/j.1748-5827.1999.tb03246.x

[21] Rundfeldt C . Quality of life of dogs with chronic epilepsy. Vet Rec. 2016;178:650‐651. 27339925 10.1136/vr.i3444

[22] Bosch G , Beerda B , Hendriks WH , van der Poel AFB , Verstegen MWA . Impact of nutrition on canine behaviour: current status and possible mechanisms. Nutr Res Rev. 2007;20:180‐194. 19079869 10.1017/S095442240781331X

[23] Packer RM , Law TH , Davies E , et al. Effects of a ketogenic diet on ADHD‐like behavior in dogs with idiopathic epilepsy. Epilepsy Behav. 2016;55:62‐68. 26773515 10.1016/j.yebeh.2015.11.014

[24] Law TH , Davies ES , Pan Y , et al. A randomised trial of a medium‐chain TAG diet as treatment for dogs with idiopathic epilepsy. Br J Nutr. 2015;114:1438‐1447. 26337751 10.1017/S000711451500313X PMC4635653

[25] Berk BA , Packer RMA , Law TH , Volk HA . Investigating owner use of dietary supplements in dogs with idiopathic epilepsy. Res Vet Sci. 2018;119:276‐284. 30064067 10.1016/j.rvsc.2018.07.004

[26] Martin K , Jackson CF , Levy RG , et al. Ketogenic diet and other dietary treatments for epilepsy. Cochrane Database Syst Rev. 2016;2:Cd001903. 26859528 10.1002/14651858.CD001903.pub3

[27] Baird JS , Ravindranath TM . Vitamin B deficiencies in a critically ill autistic child with a restricted diet. Nutr Clin Pract. 2015;30:100‐103. 25112945 10.1177/0884533614541483

[28] Baranano KW , Hartman AL . The ketogenic diet: uses in epilepsy and other neurologic illnesses. Curr Treat Options Neurol. 2008;10:410‐419. 18990309 10.1007/s11940-008-0043-8 PMC2898565

[29] Chianese R , Coccurello R , Viggiano A , et al. Impact of dietary fats on brain functions. Curr Neuropharmacol. 2017;16:1059‐1085. 10.2174/1570159X15666171017102547 PMC6120115 29046155

[30] Achanta LB , Rae CD . Beta‐hydroxybutyrate in the brain: one molecule, multiple mechanisms. Neurochem Res. 2017;42:35‐49. 27826689 10.1007/s11064-016-2099-2

[31] Augustin K , Khabbush A , Williams S , et al. Mechanisms of action for the medium‐chain triglyceride ketogenic diet in neurological and metabolic disorders. Lancet Neurol. 2018;17:84‐93. 29263011 10.1016/S1474-4422(17)30408-8

[32] Lusardi TA , Akula KK , Coffman SQ , Ruskin DN , Masino SA , Boison D . Ketogenic diet prevents epileptogenesis and disease progression in adult mice and rats. Neuropharmacology. 2015;99:500‐509. 26256422 10.1016/j.neuropharm.2015.08.007 PMC4655189

[33] Ciarlone SL , Grieco JC , D'Agostino DP , et al. Ketone ester supplementation attenuates seizure activity, and improves behavior and hippocampal synaptic plasticity in an Angelman syndrome mouse model. Neurobiol Dis. 2016;96:38‐46. 27546058 10.1016/j.nbd.2016.08.002

[34] Matthews H , Granger N , Wood J , et al. Effects of essential fatty acid supplementation in dogs with idiopathic epilepsy: a clinical trial. Vet J. 2012;191:396‐398. 21641244 10.1016/j.tvjl.2011.04.018

[35] Scorza CA , Calderazzo L , Cavalheiro EA , Cysneiros RM , Scorza FA . Sudden unexpected death in dogs with epilepsy: risks versus benefits of omega‐3 fatty acid supplementation for man's best friend. Epilepsy Behav. 2013;27:508‐509. 23619429 10.1016/j.yebeh.2013.02.021

[36] Scorza FA , Cavalheiro EA , Arida RM , et al. Positive impact of omega‐3 fatty acid supplementation in a dog with drug‐resistant epilepsy: a case study. Epilepsy Behav. 2009;15:527‐528. 19541544 10.1016/j.yebeh.2009.05.013

[37] Law TH , Volk HA , Pan Y , et al. Metabolic perturbations associated with the consumption of a ketogenic medium‐chain TAG diet in dogs with idiopathic epilepsy. Br J Nutr. 2018;120:484‐490. 10.1017/S0007114518001617 PMC6137430 30001753

[38] Patterson EEMK , Kirk CA , et al. Results of a ketogenic food trial for dogs with idiopathic epilepsy. J Vet Intern Med. 2005;19:421.

[39] Masino SA , Freedgood NR , Reichert HR , et al. Dietary intervention for canine epilepsy: two case reports. Epilepsia. 2019;4:193‐199. 10.1002/epi4.12305 PMC6398089 30868131

[40] Stassen QEM , Koskinen LLE , van Steenbeek FG , et al. Paroxysmal dyskinesia in border terriers: clinical, epidemiological, and genetic investigations. J Vet Intern Med. 2017;31:1123‐1131. 28703446 10.1111/jvim.14731 PMC5508305

[41] Lowrie M , Garden OA , Hadjivassiliou M , et al. The clinical and serological effect of a gluten‐free diet in border terriers with epileptoid cramping syndrome. J Vet Intern Med. 2015;29:1564‐1568. 26500168 10.1111/jvim.13643 PMC4895653

[42] Lowrie M , Garden OA , Hadjivassiliou M , Sanders DS , Powell R , Garosi L . Characterization of paroxysmal gluten‐sensitive dyskinesia in border terriers using serological markers. J Vet Intern Med. 2018;32:775‐781. 29424456 10.1111/jvim.15038 PMC5866963

[43] Lowrie M , Hadjivassiliou M , Sanders DS , Garden OA . A presumptive case of gluten sensitivity in a border terrier: a multisystem disorder? Vet Rec. 2016;179:573. 27784836 10.1136/vr.103910

[44] Berk BA , Packer RM , Law TH , et al. A double‐blinded randomised dietary supplement crossover trial design to investigate the short‐term influence of medium chain fatty acid (mct) supplement on canine idiopathic epilepsy: study protocol. BMC Vet Res. 2019;15:181. 31146740 10.1186/s12917-019-1915-8 PMC6543566

[45] De Risio L , Bhatti S , Munana K , et al. International veterinary epilepsy task force consensus proposal: diagnostic approach to epilepsy in dogs. BMC Vet Res. 2015;11:148. 26316175 10.1186/s12917-015-0462-1 PMC4552251

[46] Scorza CA , Cavalheiro EA , Calderazzo L , Scorza FA . Labrador retrievers and SUDEP: a simple theory that may have important applications. Epilepsy Behav. 2014;32:27‐28. 24463305 10.1016/j.yebeh.2013.12.024

[47] Rogawski MA , Johnson MR . Intrinsic severity as a determinant of antiepileptic drug refractoriness. Epilepsy Curr. 2008;8:127‐130. 18852835 10.1111/j.1535-7511.2008.00272.x PMC2566613

[48] Brodie MJ , Barry SJ , Bamagous GA , et al. Patterns of treatment response in newly diagnosed epilepsy. Neurology. 2012;78:1548‐1554. 22573629 10.1212/WNL.0b013e3182563b19 PMC3348850

[49] Packer RM , Shihab NK , Torres BB , et al. Responses to successive anti‐epileptic drugs in canine idiopathic epilepsy. Vet Rec. 2015;176:203. 10.1136/vr.102934 25564473

[50] Charalambous M , Brodbelt D , Volk HA . Treatment in canine epilepsy—a systematic review. BMC Vet Res. 2014;10:257. 25338624 10.1186/s12917-014-0257-9 PMC4209066

[51] Munana KR , Nettifee‐Osborne JA , Papich MG . Effect of chronic administration of phenobarbital, or bromide, on pharmacokinetics of levetiracetam in dogs with epilepsy. J Vet Intern Med. 2015;29:614‐619. 25711374 10.1111/jvim.12548 PMC4895521

[52] Rundfeldt C , Gasparic A , Wlaz P . Imepitoin as novel treatment option for canine idiopathic epilepsy: pharmacokinetics, distribution, and metabolism in dogs. J Vet Pharmacol Ther. 2014;37:421‐434. 24611573 10.1111/jvp.12117 PMC4280904

[53] Patsalos PN . Pharmacokinetic profile of levetiracetam: toward ideal characteristics. Pharmacol Ther. 2000;85:77‐85. 10722121 10.1016/s0163-7258(99)00052-2

[54] Thurman GD , McFadyen ML , Miller R . The pharmacokinetics of phenobarbitone in fasting and non‐fasting dogs. J S Afr Vet Assoc. 1990;61:86‐89. 2287006

[55] Gindiciosi B , Palus V , Eminaga S , Villiers E , Bruto Cherubini G . Serum bromide concentrations following loading dose in epileptic dogs. J Small Anim Pract. 2014;55:108‐111. 24433448 10.1111/jsap.12173

[56] Shaw N , Trepanier LA , Center SA , Garland S . High dietary chloride content associated with loss of therapeutic serum bromide concentrations in an epileptic dog. J Am Vet Med Assoc. 1996;208:234‐236. 8567379

[57] Maguire PJ , Fettman MJ , Smith MO , et al. Effects of diet on pharmacokinetics of phenobarbital in healthy dogs. J Am Vet Med Assoc. 2000;217:847‐852. 10997154 10.2460/javma.2000.217.847

[58] Larsen JA , Owens TJ , Fascetti AJ . Nutritional management of idiopathic epilepsy in dogs. J Am Vet Med Assoc. 2014;245:504‐508. 25148091 10.2460/javma.245.5.504

[59] Kverneland M , Tauboll E , Selmer KK , et al. Modified atkins diet may reduce serum concentrations of antiepileptic drugs. Acta Neurol Scand. 2015;131:187‐190. 25312999 10.1111/ane.12330

[60] Charalambous M , Pakozdy A , Bhatti SFM , Volk HA . Systematic review of antiepileptic drugs' safety and effectiveness in feline epilepsy. BMC Vet Res. 2018;14:64. 29499762 10.1186/s12917-018-1386-3 PMC5834883

[61] Czekaj P . Phenobarbital‐induced expression of cytochrome P450 genes. Acta Biochim pol. 2000;47:1093‐1105. 11996099

[62] Waxman DJ , Azaroff L . Phenobarbital induction of cytochrome P‐450 gene expression. Biochem J. 1992;281(Pt 3):577‐592. 1536639 10.1042/bj2810577 PMC1130728

[63] Frueh FW , Zanger UM , Meyer UA . Extent and character of phenobarbital‐mediated changes in gene expression in the liver. Mol Pharmacol. 1997;51:363‐369. 9058589

[64] Munro AW , McLean KJ , Grant JL , et al. Structure and function of the cytochrome P450 peroxygenase enzymes. Biochem Soc Trans. 2018;46:183‐196. 29432141 10.1042/BST20170218 PMC5818669

[65] Wanders RJ , Komen J , Kemp S . Fatty acid omega‐oxidation as a rescue pathway for fatty acid oxidation disorders in humans. FEBS J. 2011;278:182‐194. 21156023 10.1111/j.1742-4658.2010.07947.x

[66] Dewey CW . Anticonvulsant therapy in dogs and cats. Vet Clin North Am Small Anim Pract. 2006;36:1107‐1127. vii. 16984829 10.1016/j.cvsm.2006.05.005

[67] Levitski RE , Trepanier LA . Effect of timing of blood collection on serum phenobarbital concentrations in dogs with epilepsy. J Am Vet Med Assoc. 2000;217:200‐204. 10909458 10.2460/javma.2000.217.200

[68] Monteiro R , Anderson TJ , Innocent G , Evans NP , Penderis J . Variations in serum concentration of phenobarbitone in dogs receiving regular twice daily doses in relation to the times of administration. Vet Rec. 2009;165:556‐558. 19897869 10.1136/vr.165.19.556

[69] Li J , O'Leary EI , Tanner GR . The ketogenic diet metabolite beta‐hydroxybutyrate (beta‐HB) reduces incidence of seizure‐like activity (SLA) in a Katp‐ and GABAb‐dependent manner in a whole‐animal *Drosophila melanogaster* model. Epilepsy Res. 2017;133:6‐9. 28395176 10.1016/j.eplepsyres.2017.04.003

[70] Si J , Wang S , Liu N , et al. Anticonvulsant effect of exogenous beta‐hydroxybutyrate on kainic acid‐induced epilepsy. Exp Ther Med. 2017;14:765‐770. 28672997 10.3892/etm.2017.4552 PMC5488665

[71] Hu E , Du H , Zhu X , et al. Beta‐hydroxybutyrate promotes the expression of BDNF in hippocampal neurons under adequate glucose supply. Neuroscience. 2018;386:315‐325. 29966721 10.1016/j.neuroscience.2018.06.036

[72] Kovacs Z , D'Agostino DP , Ari C . Anxiolytic effect of exogenous ketone supplementation is abolished by adenosine A1 receptor inhibition in Wistar Albino Glaxo/Rijswijk rats. Front Behav Neurosci. 2018;12:29. 29520223 10.3389/fnbeh.2018.00029 PMC5827672

[73] Clanton RM , Wu G , Akabani G , Aramayo R . Control of seizures by ketogenic diet‐induced modulation of metabolic pathways. Amino Acids. 2017;49:1‐20. 27683025 10.1007/s00726-016-2336-7

[74] Boison D . New insights into the mechanisms of the ketogenic diet. Curr Opin Neurol. 2017;30:187‐192. 28141738 10.1097/WCO.0000000000000432 PMC5409832

[75] Chang P , Augustin K , Boddum K , et al. Seizure control by decanoic acid through direct AMPA receptor inhibition. Brain J Neurol. 2015;139:431‐443. 10.1093/brain/awv325 PMC4805082 26608744

[76] Augustin K , Williams S , Cunningham M , et al. Perampanel and decanoic acid show synergistic action against AMPA receptors and seizures. Epilepsia. 2018;59:e172‐e178. 30324610 10.1111/epi.14578

[77] Hughes SD , Kanabus M , Anderson G , et al. The ketogenic diet component decanoic acid increases mitochondrial citrate synthase and complex I activity in neuronal cells. J Neurochem. 2014;129:426‐433. 24383952 10.1111/jnc.12646

[78] Schuck PF , Ferreira Gda C , Tonin AM , et al. Evidence that the major metabolites accumulating in medium‐chain acyl‐CoA dehydrogenase deficiency disturb mitochondrial energy homeostasis in rat brain. Brain Res. 2009;1296:117‐126. 19703432 10.1016/j.brainres.2009.08.053

[79] Wlaz P , Socala K , Nieoczym D , et al. Acute anticonvulsant effects of capric acid in seizure tests in mice. Prog Neuropsychopharmacol Biol Psychiatry. 2015;57:110‐116. 25445478 10.1016/j.pnpbp.2014.10.013

[80] Chang P , Orabi B , Deranieh RM , et al. The antiepileptic drug valproic acid and other medium‐chain fatty acids acutely reduce phosphoinositide levels independently of inositol in Dictyostelium. Disc Model Mech. 2012;5:115‐124. 10.1242/dmm.008029 PMC3255550 21876211

[81] Chang P , Terbach N , Plant N , Chen PE , Walker MC , Williams RSB . Seizure control by ketogenic diet‐associated medium chain fatty acids. Neuropharmacology. 2013;69:105‐114. 23177536 10.1016/j.neuropharm.2012.11.004 PMC3625124

[82] Chang P , Zuckermann AM , Williams S , et al. Seizure control by derivatives of medium chain fatty acids associated with the ketogenic diet show novel branching‐point structure for enhanced potency. J Pharmacol Exp Ther. 2015;352:43‐52. 25326131 10.1124/jpet.114.218768

[83] Khabbush A , Orford M , Tsai YC , et al. Neuronal decanoic acid oxidation is markedly lower than that of octanoic acid: a mechanistic insight into the medium‐chain triglyceride ketogenic diet. Epilepsia. 2017;58:1423‐1429. 28682459 10.1111/epi.13833

[84] Haidukewych D , Forsythe WI , Sills M . Monitoring octanoic and decanoic acids in plasma from children with intractable epilepsy treated with medium‐chain triglyceride diet. Clin Chem. 1982;28:642‐645. 7074833

[85] Andersen G , Souci SW , Fachmann W , et al. Lebensmitteltabelle für die Praxis: Der Kleine Souci‐Fachmann‐Kraut. Stuttgart, Germany: Wissenschaftliche Verlagsgesellschaft Stuttgart; 2011.

[86] Souci SW , Fachmann, W , Kraut, H . Food Composition and Nutrition Tables. Stuttgart, Germany: Wissenschaftliche Verlagsgesellschaft Stuttgart; 2016.

[87] Hand MS , Thatcher CD , Remillard RL , Roudebush P . Small Animal Clinical Nutrition. Portland, OR: Mark Morris Institute; 2010.

[88] Matulka RA , Thompson DV , Burdock GA . Lack of toxicity by medium chain triglycerides (MCT) in canines during a 90‐day feeding study. Food Chem Toxicol. 2009;47:35‐39. 19135768 10.1016/j.fct.2008.06.080

[89] Beynen AC , Kappert HJ , Lemmens AG , van Dongen AM . Plasma lipid concentrations, macronutrient digestibility and mineral absorption in dogs fed a dry food containing medium‐chain triglycerides. J Anim Physiol Anim Nutr. 2002;86:306‐312. 10.1046/j.1439-0396.2002.00387.x 12452972

[90] Hall JA , Jewell DE . Feeding healthy beagles medium‐chain triglycerides, fish oil, and carnitine offsets age‐related changes in serum fatty acids and carnitine metabolites. PLoS One. 2012;7:e49510. 23145181 10.1371/journal.pone.0049510 PMC3492282

[91] James FE , Mansfield CS , Steiner JM , Williams DA , Robertson ID . Pancreatic response in healthy dogs fed diets of various fat compositions. Am J Vet Res. 2009;70:614‐618. 19405900 10.2460/ajvr.70.5.614

[92] Council NR . Nutrient Requirements of Dogs and Cats. Washington, DC: National Academies Press; 2006.

[93] Gravel J , Opatrny L , Shapiro S . The intention‐to‐treat approach in randomized controlled trials: are authors saying what they do and doing what they say? Clinical Trials. 2007;4:350‐356. 17848496 10.1177/1740774507081223

