# Erratum for “A multicenter randomized controlled trial of medium‐chain triglyceride dietary supplementation on epilepsy in dogs”

**DOI:** 10.1111/jvim.17140

**Published:** 2024-07-04

Berk BA, Law TH, Packer RMA, et al. A multicenter randomized controlled trial of medium‐chain triglyceride dietary supplementation on epilepsy in dogs. *J Vet Intern Med*. 2020;34(3):1248‐1259. doi:10.1111/jvim.15756

The conflict of interest declaration was missing information about two authors. The missing information is shown below:

Andrea Tipold serves as Associate Editor for the Journal of Veterinary Internal Medicine. She was not involved in review of this manuscript.

Holger Volk in last 5 years served as contract researcher for: Nestle 2012‐2014 and 2017‐2020—dietary modification of epilepsy in dogs; Boehringer Ingelheim 2014‐2015—investigating the effects of imepitoin behavioral, physiologic and owner reported indicators of anxiety in dogs treated for idiopathic epilepsy; CASE BBSRC PhD studentship 2012‐2016—metabolic profiling of epilepsy in dogs; American Kennel Club American Health Foundation 2016‐2020—Investigating the Effect of a Ketogenic Medium Chain Triglycerides Supplement on the treatment of Canine Idiopathic Epilepsy and its behavioral comorbidities; BBSRC 2017‐2020—Investigating the relationship between epilepsy, drug‐resistance and affective disorders in the domestic dog BB/P001874/1; provided continuous education for veterinary surgeons sponsored by Nestle, Boehringer Ingelheim and Virbac in the last 5 years.

The authors did not allow any bias from any of these conflicts of interest to affect the design, analysis or writing of the paper.

