# Supplementary material for: A multicenter randomized controlled trial of medium‐chain triglyceride dietary supplementation on epilepsy in dogs
Source: J Vet Intern Med. 2020 Apr 15;34(3):1248–59. doi: 10.1111/jvim.15756 (PMC7255680; doi:10.1111/jvim.15756)
Supplement: Supplementary file 1 — Table S1. Adverse events recorded for dogs during the dietary supplementation with the control and MCT‐DS Table S2. Concomitant treatment Table S3. Age, weight, sex, neuter status, breed and base diet composition of the all dogs Table S4. Antiepileptic drug medication regimes of each individual dog included in this study Table S5. Trial diet composition—the average macronutrient profile of the base diet Table S6. Overview of seizure frequency per month and seizure day frequency per month in each dog during the control DS and MCT‐DS Table S7. Correlation coefficient analysis between seizure frequency reduction and seizure day frequency reduction with age, weight and BHB concentrations [file JVIM-34-1248-s001.pdf]

Supplementary Table 1. **Adverse events recorded for dogs during the dietary supplementation with the control and MCT-DS.** Details of the adverse event includes: a description of the adverse event; length of adverse event recorded in days; severity of adverse event recorded using 1=mild, 2=moderate, 3=severe and 4=serious; frequency of adverse event measured using 1=once, 2=occasionally, 3=regularly and 4=ongoing; causality of adverse event recorded using 1=disease diagnosed before enrolment, 2=disease diagnosed after enrolment, 3=unknown; concomitant treatment noted as 1=none and 2=yes and final outcomes consisted of 1=resolved without further effects, 2=resolved with further effects, 3=unchanged, 4=euthanasia and 5=death. Adverse events were recorded over a period of 3 months (±2 days) for the control DS and 3 months (±2 days) for the MCT-DS. In reference of the study objectives, seizure occurrences during the study participation were not defined as an adverse event.

	**Cases**	**Adverse effects**	**Length of effect (days)**	**Severity**	**Frequency**	**Causality**	**Treatment**	**Outcome**
**C**	SCN3	Urolithiasis, bladder stones	Ongoing	1	1	2	2	1
**O N T**	SCN8	Adverse effects by apomorphine injection due to single Phenobarbital overdose	2	2	1	2	2	1
**R O**	SCN12	Azotaemia	21	no clinical signs	1	2	1	1
**L**	SCN18	Urinary tract infection	7	1	1	2	2	1
*****		Malassezia ear infection	Ongoing	1	1	2	2	3
**D**		Linear foreign body obstruction	75	2	1	2	2	1

**S**	SCN20	Dog Attack	8	1	1	2	2	1
**MCT*DS**	SCN3	Diarrhoea	4	1	1	2	2	1
	SCN6	Lethargy, restlessness	4	1	1	2	1	1
	SCN10	Worsening of pre-existing incontinence - chronic *E. coli* urinary tract infection	40	1	2	1	2	1
	SCN15	Vomiting series unknown cause	7	1	1	2	2	1
	SCN17	Yeast ear infection	3	1	1	2	2	1
		Malassezia skin infection, recurrent	Ongoing	1	1	2	2	1
	SCN18	Worsening of pre-existing hip dysplasia, coxarthrosis	Ongoing	2	3	2	2	3
	SCN19	Dog attack	2	1	1	1	2	1
	SCN20	Urinary tract infection	8	1	1	2	2	1

Supplementary Table 2. Concomitant treatment recorded for dogs during the dietary supplementation with the control and MCT-DS detailing indications for treatment, products used and length of treatments. Concomitant treatments were recorded over a period of 3 months (±2 days) for the control DS and 3 months (±2 days) for the MCT-DS.

	Cases	Indication for treatment	Products	Length of treatment (days)
	**SCN3**	Cystitis	Marbofloxacin	7
		Cystitis	Meloxicam	7
		Sonographic investigation of the bladder	Isoflurane, buprenorphine, propofol	1
		Surgical bladder stone removal	Isoflurane, buprenorphine, propofol, meloxicam, enrofloxacin	1
		Postoperative care	Marbofloxacin	7
**C**	**SCN6**	Deflea/dewormer (3 monthly)	Imidacloprid, moxidectin combination spot on, praziquantel, pyrantel pamoate combination tablet	1
**O N**	**SCN8**	Accidential phenobarbital overdose	Apomorphine, 2 mg	1
**T R**		Deflea/dewormer (3 monthly)	Imidacloprid, moxidectin combination spot on, milbemycine oxime	1

O<br>L	**SCN9**	Ear infection	Marbofloxacin, clotrimazole, dexamethason acetate drops	10
*	**SCN10**	Dewormer (3 monthly)	Milbemycin oxime	1
D		Pain relief due to hip dysplasia	Meloxicam	Ongoing
S		Incontinence	Enurance	Ongoing
		Incontinence	Estriol	Ongoing
		Gastritis, hyperaciditiy	Ranitidine	Ongoing
	**SCN12**	Sonographic kidney investigation	Medetomidine, atipamezol, propofol, isoflurane	1
		Deflea/dewormer (3 monthly)	Imidacloprid, moxidectin combination spot on,	1
	**SCN15**	Vaccination	Nobivac l4 (leptospirosis)	1
	**SCN16**	Deflea/dewormer (3 monthly)	Imidacloprid, moxidectin combination spot on	1
		Eye dryness	Cyclosporine eye ointment	Ongoing
	**SCN18**	Malassezia Ear Infection	Terbinafine, florfenicol, betamethasone acetate combination drops, chlorhexidine	1
		Cystitis	Amoxicillin, clavulanic acid	7
		Deflea/dewormer (3 monthly)	Fluralaner, milbemycin oxime	1
		Ear infection	Osurnia	5

		Ear infection	Gentamicin, clotrimazole	7
		Linear foreign body	Buprenorhine, midazolam, propofol, fentanyl, methadone	1
		Linear foreign body postoperative care	Cefuroxime, paracetamol, maropitant, buprenorphine	7
**C**	**SCN19**	Degenerative Joint Disease	Meloxicam	Ongoing
**O**	**SCN20**	Deflea/dewormer (3 monthly)	Afoxoclaner	1
**N**		Puncture wound	Meloxicam	7
**T**		Puncture wound	Amoxicillin	7
**R**	**SCN23**	Coughing	Amoxicillin	1
**O**		Deflea/dewormer (3 monthly)	Praziquantel, fipronil and (s)-methoprene	1
**L**	**SCN26**	Chocolate intake	Apomorphine	1
*****		Chocolate intake	Metoclopramide	1
**D**	**SCN28**	Extraction of injured tooth	Medetomidine, atipamezol, meloxicam	1
**S**		Pain relief	Meloxicam	7
	**SCN29**	Hypothyreosis	L-thyroxine	Ongoing
	**SCN30**	Inflammatory bowel disease	Cefalexin	Ongoing

		Gall bladder obstruction	Ursocholdecochylacid	Ongoing
		Bradycardia of unknown origin	Theophylline	Ongoing
	**SCN34**	Vomiting and nausea	Famotidine	3
	**SCN35**	Urinary tract infection	Amoxicillin	14
		Urinary tract infection	Amoxicillin and clavulanic acid	14
**MCT*DS**	**SCN1**	Corn Treatment at left hindlimb	Natural ointment/ remedies	14
	**SCN3**	Urinary tract infection	Marbofloxacin	7
		Urinary tract infection	Meloxicam	7
	**SCN8**	Deflea/dewormer (3 monthly)	Fluralaner, meloxicam	1
	**SCN9**	Anal gland infection	Amoxicillin, meloxicam	7
	**SCN10**	Sedation for cystocentesis	Medetomidine, butorphanol, atipamezole	1
		E.Coli urinary tract infection	Trimetophrime/ sulfondamide (tso)	20
		E.Coli urinary tract infection	Ampicilline	20
		Pain relief, hip dysplasia	Meloxicam	Ongoing
		Incontinence	Enurance	Ongoing
		Incontinence	Estriol	Ongoing
		Gastritis, hyperaciditiy	Ranitidine	Ongoing

M C T * D S	**SCN11**	Paw infection	Amoxicillin and clavulanic acid	7
		Paw infection	Meloxicam	10
	**SCN12**	Deflea/dewormer (3 monthly)	Imidacloprid, moxidectin combination spot on	1
	**SCN15**	Gastritis	Ranitidine	1
		Deflea/dewormer (3 monthly)	Fluralaner, milbemycin oxime	1
		Vaccination	Nobivac kc25	1
	**SCN16**	Vaccination	L + kc	1
	**SCN17**	Ear infection	Terbinafine, florfenicol, betamethasone acetate combination drops, edta based cleaning solution	1
		Hip exam	Acepromazine, butorphanol, propofol	1
	**SCN18**	Hip exam	Acepromazine, medetomidine, ketamine, propofol, isoflurane, comfortan, antisedan	1
		Lameness	Firocoxib	Ongoing
	**SCN19**	Dog Attack	Amoxcillin	1
	**SCN23**	Immune stimulation	Parapoxvirus ovis d 1701	1
		Deflea/dewormer (3 monthly)	Praqziquantel, fenbendazole, fipronil and (s)-methoprene	1

	**SCN24**	Deflea/dewormer (3 monthly)	Imidacloprid, moxidectin combination spot on, milbemycin oxime	1
	**SCN29**	Hypothyreosis	L-thyroxine	ongoing
	**SCN33**	Otitis externa	Cotrimazole, marbofloxacin, dexamethasone acetate drops	5
	**SCN34**	New Year’s Eve	1 drop cbd oil, 5%, unknown brand	1

Supplementary Table 3. Age, weight, sex, neuter status, breed and base diet composition of the all dogs included in this study recruited at six different study sides (1 = Royal Veterinary College, Department of Clinical Science and Services (CSS), Hatfield, United Kingdom; 2 = Pride Veterinary Centre, Riverside Road, Pride Park, Derby, UK; 4 = Tierarztpraxis , Dr. A. Bathen-Nöthen ,Hatzfeldstraße, Cologne, Germany; 5 = Faculty of Veterinary Medicine, Dep. of Equine and Small Animal Medicine, Helsinki, Finland; 6 = Klinik für Kleintiere, Stiftung Tierärztliche Hochschule Hannover, Bünteweg, Hanover, Germany; 7 = Tierarztpraxis Strassenheim, Ortsstraße, Mannheim, Germany)

**Dog SCN**	**Study Side**	**Dog breed**	**AGE (years.months)**	**Weight (KG)**	**Sex (M/F)**	**Neuter status (Y/N)**	**Base diet composition**
SCN1	1	Whippet	6.7	9.4	FEMALE	Y	Raw Food
SCN2	1	Border Collie	2.2	18.5	FEMALE	N	Dry Food + Raw Food
SCN3	1	Bernese Mountain Dog	2.1	31.5	FEMALE	Y	Dry Food
SCN5	1	German Shepherd Dog	6.5	51.8	MALE	Y	Home Cooked F + Raw Food
SCN6	1	Cross Breed	6.4	23.8	MALE	Y	Dry Food (Prescp. Diet)
SCN8	1	Border Collie	2.11	14.8	FEMALE	Y	Dry Food
SCN9	1	Chihuahua (Smooth Coat)	7.6	4.7	FEMALE	Y	Dry Food + Home Cooked F
SCN10	1	Slovakian Rough Haired Pointer	8.8	32.3	FEMALE	Y	Dry Food + Home Cooked F
SCN11	1	Keeshond	6.3	24.3	FEMALE	N	Raw Food

SCN12	1	Cross Breed	5.3	42.3	MALE	Y	Dry Food
SCN15	1	Border Terrier	6.3	9.6	MALE	Y	Dry Food (Prescp. Diet)
SCN16	1	Hungarian Vizsla	2.2	28.2	MALE	Y	Dry Food
SCN17	2	Cross Breed	4	44.9	MALE	Y	Dry Food
SCN18	2	Basset Griffon Vendeen (Petit)	7.2	17.9	MALE	Y	Dry Food (Prescp. Diet)
SCN19	2	Retriever (Labrador)	9.10	37.2	MALE	Y	Dry Food
SCN20	2	Dogue de Bordeaux	4.2	47.7	FEMALE	Y	Dry Food + Raw Food
SCN23	7	Crossbreed	10.3	10.0	FEMALE	Y	Dry Food + Wet Food
SCN24	7	Samoyed	10.1	17.3	FEMALE	Y	Dry Food
SCN25	7	Crossbreed	10.11	29.7	MALE	N	Dry Food + Wet Food
SCN26	7	Australian Shepherd	3.1	27.0	MALE	Y	Raw Food
SCN27	4	Crossbreed	2.7	19.2	FEMALE	Y	Dry Food
SCN28	4	Crossbreed	8.1	23.8	MALE	Y	Dry Food + Raw Food
SCN29	4	Retriever (Golden)	4.11	30.7	MALE	N	Dry Food + Raw Food
SCN30	4	Staffordshire Bull Terrier	9.11	28.0	MALE	Y	Dry Food + Wet Food
SCN32	6	Border Collie	8.11	27.8	MALE	N	Home Cooked F + Wet Food
SCN33	6	Samoyed	2	55.3	MALE	N	Dry Food

SCN34	6	Crossbreed	5.3	23.5	MALE	Y	Dry Food
SCN35	5	Cairn Terrier	6.5	9.6	FEMALE	Y	Dry Food + Home Cooked F

Supplementary Table 4. Antiepileptic drug medication regimes of each individual dog included in this study. While chronic medication was administered, pulse therapy was individually used according to scientifically proven protocols at the onset of seizing. (1 = Royal Veterinary College, Department of Clinical Science and Services (CSS), Hatfield, United Kingdom; 2 = Pride Veterinary Centre, Riverside Road, Pride Park, Derby, UK; 4 = Tierarztpraxis, Dr. A. Bathen-Nöthen ,Hatzfeldstraße, Cologne, Germany; 5 = Faculty of Veterinary Medicine, Dep. of Equine and Small Animal Medicine, Helsinki, Finland; 6 = Klinik für Kleintiere, Stiftung Tierärztliche Hochschule Hannover, Bünteweg, Hanover, Germany; 7 = Tierarztpraxis Strassenheim, Ortsstraße, Mannheim, Germany)

**Dog SCN**	**Study Side**	**Chronic medication**						**Pulse therapy**	
		**Phenobarbital**	**Potassium Bromide**	**Levetiracetam**	**Imepitoin**	**Retigabine**	**Gabapentin**	**Levetiracetam**	**Diazepam**
SCN1	1	X	X	X					
SCN2	1	X	X					X	X
SCN3	1	X	X	X				X	X
SCN5	1	X			X		X	X	X
SCN6	1	X	X	X					
SCN8	1	X	X	X					
SCN9	1	X		X	X				

SCN10	1	X	X						X
SCN11	1		X	X					
SCN12	1				X				
SCN15	1				X				X
SCN16	1	X	X	X					X
SCN17	2	X	X						X
SCN18	2	X	X	X					X
SCN19	2	X		X					
SCN20	2	X	X	X					
SCN23	7	X	X						X
SCN24	7	X		X		X			
SCN25	7	X	X	X					
SCN26	7	X	X		X				X
SCN27	4	X	X	X					X
SCN28	4	X	X		X				
SCN29	4	X	X	X					
SCN30	4	X	X	X					

SCN32	6	X		X					X
SCN33	6	X	X	X					X
SCN34	6	X		X					
SCN35	5	X	X						
N		25	20	18	6	1	1	3	12
%		89	71	64	21	4	4	11	43

Supplemental table 5. Trial diet composition – The average macronutrient profile of the base diet from 19 dogs of the trial population calculated from analytical data provided by each company.

	% in 100 g/ 1000 g						
**DOG**	**Crude Protein**	**Crude Fat**	**Crude Fibers**	**Crude Ash**	**Moisture**	**Calcium**	**Phosphorus**
**SCN3**	21,00%	12,00%	2,50%	6,00%	8,50%	1,04%	0,73%
**SCN5**	16,00%	10,25%	2,00%	4,50%	43,25%	1,40%	0,72%
**SCN6**	26,00%	11,00%	16,00%	8,00%	10,00%	0,80%	0,60%
**SCN8**	18,50%	8,00%	2,50%	7,50%	8,00%	2,06%	1,05%
**SCN9**	30,00%	11,00%	6,80%	5,10%	8,00%	0,82%	0,64%
**SCN10**	25,00%	14,00%	8,00%	3,00%	8,00%	1,60%	1,00%
**SCN12**	26,20%	16,00%	2,60%	5,50%	8,00%	0,95%	0,75%
**SCN15**	22,70%	12,80%	1,30%	6,20%	10,00%	0,99%	0,72%
**SCN16**	18,50%	7,50%	2,50%	7,00%	8,00%	1,30%	0,87%
**SCN18**	17,39%	15,76%	1,30%	5,50%	10,00%	0,95%	0,66%

**SCN19**	20,00%	7,50%	3,00%	5,00%	10,00%	N/A	N/A
**SCN20**	18,00%	7,50%	3,50%	7,00%	10,00%	0,90%	0,60%
**SCN24**	20,00%	8,00%	3,50%	7,50%	10,00%	1,20%	0,80%
**SCN27**	26,00%	16,00%	2,00%	7,90%	10,00%	1,50%	1,00%
**SCN28**	22,00%	12,00%	3,00%	7,00%	10,00%	1,50%	1,00%
**SCN30**	16,50%	8,75%	1,70%	4,45%	42,00%	0,75%	0,50%
**SCN32**	26,00%	16,00%	9,50%	3,50%	8,00%	2,30%	1,50%
**SCN33**	23,00%	16,50%	1,20%	6,50%	10,00%	0,30%	0,25%
**SCN34**	22,70%	12,80%	1,30%	6,20%	10,00%	0,99%	0,72%
	**Crude Protein**	**Crude Fat**	**Crude Fibers**	**Crude Ash**	**Moisture**	**Calcium**	**Phosphorus**
**AVERAGE:**	**21,87%**	**11,76%**	**3,91%**	**5,97%**	**12,72%**	**1,19%**	**0,78%**

Supplementary Table 6. Overview of seizure frequency per month and seizure day frequency per month in each dog during the control DS and MCT-DS respectively (n=28). Overall response is represented by the percentage change between outcome variables respectively. Wilcoxon matched paired t-tests were used to make comparisons between diet groups. Statistical significances between diets are presented as p-values and highlighted in blue.

	**Seizure frequency per month (p=0.015)**				**Seizure day frequency per month (p=0.010)**		
**DOG SCN**	**Control**	**MCT-DS**	**Change (%)**	**DOG SCN**	**Control**	**MCT-DS**	**Change (%)**
SCN01	2,667	2,386	-11	SCN01	2,667	2,386	-11
SCN02	1,333	0,667	-50	SCN02	1,333	0,667	-50
SCN03	3,667	3,956	8	SCN03	3,333	2,967	-11
SCN05	1,957	0,659	-66	SCN05	1,630	0,659	-60
SCN06	1,364	0,957	-30	SCN06	0,682	0,957	40
SCN08	2,637	2,308	-13	SCN08	1,978	1,978	0
SCN09	10,449	5,604	-46	SCN09	7,416	5,604	-24
SCN10	0,000	0,319	100	SCN10	0,000	0,319	100

SCN11	4,286	0,000	-100	SCN11	1,978	0,000	-100
SCN12	4,045	6,333	57	SCN12	2,022	1,333	-34
SCN15	0,326	0,000	-100	SCN15	0,326	0,000	-100
SCN16	3,409	3,587	5	SCN16	1,364	1,957	43
SCN17	1,000	1,000	0	SCN17	1,000	1,000	0
SCN18	2,667	3,000	13	SCN18	2,000	2,000	0
SCN19	2,667	2,000	-25	SCN19	2,000	1,333	-33
SCN20	2,000	2,000	0	SCN20	2,000	2,000	0
SCN23	1,978	2,637	33	SCN23	1,978	2,637	33
SCN24	4,945	2,637	-47	SCN24	3,297	1,978	-40
SCN25	3,191	1,765	-45	SCN25	1,596	1,412	-12
SCN26	4,945	4,719	-5	SCN26	1,648	2,022	23
SCN27	9,886	6,667	-33	SCN27	2,386	2,000	-16
SCN28	2,333	2,386	2	SCN28	2,000	1,705	-15
SCN29	4,091	4,000	-2	SCN29	2,727	1,667	-39
SCN30	4,000	1,705	-57	SCN30	2,333	1,364	-42
SCN32	5,667	5,795	2	SCN32	2,667	2,386	-11

SCN33	4,773	4,000	-16	SCN33	3,750	4,000	7
SCN34	2,667	2,667	0	SCN34	1,667	1,667	0
SCN35	2,667	2,637	-1	SCN35	1,333	1,319	-1

Supplemental table 7. Correlation coefficient analysis between seizure frequency reduction and seizure day frequency reduction with age, weight and BHB concentrations (n=28)

**Variables compared in Pearson's correlation coefficient analysis**	**Statistical significance (p value)**	**Pearson's correlation (r value)**
Age vs Seizure frequency per month reduction	0.822	-0.045
Age vs Seizure day frequency per month reduction	0.355	-0.185
Age vs. Cluster Seizure Status (MCT-DS)	0.893	0.027
Age vs. Cluster Seizure Status (Control-DS)	0.325	-0193
Pre BHB DS independent vs. total seizures per dietary intervention period	0.084	-0.187
Post BHB DS independent vs. total seizure per dietary intervention period	0.064	-0.206
BHB (pre-post difference) vs. total seizure per dietary intervention period	0.222	-0.104
Pre BHB (MCT-DS) vs Seizure frequency per month (MCT-DS)	0.359	-0.180
Pre BHB (MCT-DS) vs Seizure days per month (MCT-DS)	0.769	-0.058

Post BHB (MCT-DS) vs Seizure frequency per month (MCT-DS)	0.506	-0.131
Post BHB (MCT-DS) vs Seizure days per month (MCT-DS)	0.824	0.044
Pre BHB (Control-DS) vs Seizure frequency per month (Control-DS)	0.370	-0.176
Pre BHB (Control-DS) vs Seizure days per month (Control-DS)	0.844	-0.039
Post BHB (Control-DS) vs Seizure frequency per month (Control-DS)	0.194	-0.253
Post BHB (Control-DS) vs Seizure days per month (Control-DS)	0.679	-0.082
Pre-Post BHB difference (MCT-DS) vs Seizure frequency per month reduction	0.188	-0.262
Pre-Post BHB difference (MCT-DS) vs Seizure day frequency per month reduction	0.388	-0.173
Pre-Post BHB difference (Control-DS) vs Seizure frequency per month reduction	0.429	-0.159
Pre-Post BHB difference (Control -DS) vs Seizure day frequency per month reduction	0.223	-0.243
Pre-Post BHB difference (MCT-DS – Control DS) vs Seizure frequency per month reduction	0.508	-0.133
Pre-Post BHB difference (MCT-DS – Control DS) vs Seizure day frequency per month reduction	0.851	0.038
Weight (average) vs Seizure frequency per month reduction	0.446	0.153
Weight (average) vs Seizure day frequency per month reduction	0.893	0.027
Weight (MCT-DS) vs Seizure frequency per month (MCT-DS)	0.894	-0.026
Weight (MCT-DS) vs Seizure day frequency per month (MCT-DS)	0.533	-0.123
Weight (MCT-DS) vs. Cluster Seizure Status (MCT-DS)	0.590	-0.106

Weight (Control-DS) vs Seizure frequency per month (Control-DS)	0.338	-0.188
Weight (Control-DS) vs Seizure day frequency per month (Control-DS)	0.383	-0.171
Weight (Control-DS) vs Cluster Seizure Status (Control-DS)	0.410	0.162
Pre BHB (MCT-DS) vs Cluster Seizure Status (MCT-DS)	0.236	-0.231
Post BHB (MCT-DS) vs Cluster Seizure Status (MCT-DS)	0.669	-0.084
Pre-Post BHB difference (MCT-DS) vs Cluster Seizure Status (MCT-DS)	0.092	-0.325
Pre BHB (Control-DS) vs Cluster Seizure Status (Control-DS)	0.922	-0.019
Post BHB (Control-DS) vs Cluster Seizure Status (Control-DS)	0.862	-0.034
Pre-Post BHB difference (Control-DS) vs Cluster Seizure Status (Control-DS)	0.993	0.002
